# Supplementary material for: Improving Methanol Utilization by Reducing Alcohol Oxidase Activity and Adding Co-Substrate of Sodium Citrate in Pichia pastoris
Source: J Fungi (Basel). 2023 Mar 29;9(4):422. doi: 10.3390/jof9040422 (PMC10142128; doi:10.3390/jof9040422)
Supplement: Supplementary file 1 [file jof-09-00422-s001.zip › jof-2250804-supplementary.pdf]

Supplementary Table S1 Differentially expressed genes identified by RNA-seq

Gene name	Gene description	Log2FC(A1_464/WT)	Padjust	Regulate
PAS_FragB_0004	Small nuclear ribonucleoprotein G	1.007009	3.22E-17	up
PAS_FragB_0009	H subunit of the mitochondrial glycine decarboxylase complex	1.014027	4.73E-33	up
PAS_FragB_0014	Mitochondrial DNA replication protein	0.665449	7.51E-13	up
PAS_FragB_0018	Hypothetical protein	0.805499	0.070077	up
PAS_FragB_0023	Vitamin H transporter 1	0.954501	4.31E-09	up
PAS_FragB_0029	hypothetical protein	0.849313	1.69E-22	up
PAS_FragB_0039	DNA-dependent ATPase	0.91668	2.29E-18	up
PAS_FragB_0041	hypothetical protein	0.673143	6.23E-14	up
PAS_FragB_0042	Adrenodoxin homolog, mitochondrial	0.748243	7.09E-08	up
PAS_FragB_0048	Protein ROT1	1.016748	5.56E-24	up
PAS_FragB_0050	Uncharacterized transporter	1.474097	7.56E-28	up
PAS_FragB_0051	Hypothetical protein	1.085757	8.01E-17	up
PAS_FragB_0052	Translational elongation factor EF-1 alpha	1.149689	5.37E-43	up
PAS_FragB_0054	WD repeat-containing protein	0.921295	3.26E-15	up
PAS_FragB_0055	ubiquitin-conjugating enzyme	0.63512	2.81E-06	up
PAS_FragB_0074	hypothetical protein	1.173946	2.77E-37	up
PAS_FragD_0020	Hypothetical protein	0.641126	3.50E-11	up
PAS_FragD_0024	Vacuolar H+/Ca2+ exchanger involved in control of cytosolic Ca2+ concentration	0.774729	2.22E-20	up
PAS_FragD_0025	G1 cyclin, associates with Pho85p cyclin-dependent kinase (Cdk)	0.836422	5.04E-16	up
PAS_c121_0012	Hypothetical protein	0.726047	2.49E-13	up
PAS_c121_0014	mitochondrial 54S ribosomal protein YmL28	1.118668	9.05E-14	up
PAS_c131_0011	60S ribosomal protein L14	0.654512	4.83E-17	up
PAS_chr1-1_0002	Plasma membrane H+-ATPase, pumps protons out of the cell	0.724581	3.43E-19	up
PAS_chr1-1_0028	Glycerol proton symporter of the plasma membrane, subject to glucose-induced inactivation	0.79819	8.89E-12	up
PAS_chr1-1_0037	Hypothetical protein	0.824553	3.63E-21	up
PAS_chr1-1_0049	Ribonuclease H2 catalytic subunit, removes RNA primers during Okazaki fragment synthesis	0.892167	1.32E-17	up
PAS_chr1-1_0050	Dihydrolipoamide acetyltransferase component (E2) of pyruvate dehydrogenase complex	0.842026	8.07E-21	up
PAS_chr1-1_0072	Fructose 1,6-bisphosphate aldolase, required for glycolysis and gluconeogenesis	0.733655	5.93E-25	up
PAS_chr1-1_0076	40S ribosomal protein S22	0.68876	2.26E-05	up
PAS_chr1-1_0085	Hypothetical protein	1.596055	1.68E-14	up
PAS_chr1-1_0099	Core subunit of the ubiquinol-cytochrome c reductase complex (bc1 complex)	0.818936	4.37E-22	up
PAS_chr1-1_0107	NADP(+)-dependent glutamate dehydrogenase	0.709067	1.28E-20	up
PAS_chr1-1_0111	Protein involved in negative regulation of transcription of iron regulon	0.728506	1.31E-17	up
PAS_chr1-1_0118	Hypothetical protein	0.595127	5.84E-15	up
PAS_chr1-1_0123	Putative sensor/transporter protein involved in cell wall biogenesis	0.643101	1.26E-12	up
PAS_chr1-1_0126	Hypothetical protein	0.644746	1.35E-05	up
PAS_chr1-1_0127	Hypothetical protein	1.208273	2.01E-36	up
PAS_chr1-1_0129	Putative protein of unknown function	0.595971	6.65E-15	up
PAS_chr1-1_0130	Daughter cell-specific secreted protein with similarity to glucanases, endo-1,3-beta-glucanase	0.624163	9.09E-11	up
PAS_chr1-1_0132	Purine nucleoside phosphorylase, specifically metabolizes inosine & guanosine nucleosides	0.905213	1.26E-23	up
PAS_chr1-1_0135	Hypothetical protein	0.616349	1.73E-07	up
PAS_chr1-1_0137	Hypothetical protein	0.708184	0.056009	up
PAS_chr1-1_0141	Non-essential protein of unknown function	0.745679	8.15E-14	up
PAS_chr1-1_0157	Protein of unknown function, expression is sensitive to nitrogen catabolite repression	1.010511	5.46E-29	up

PAS_chr1-1_0158	Putative transmembrane protein involved in export of ammonia, a starvation signal	0.962978	2.93E-19	up
PAS_chr1-1_0159	Protein of unknown function, required for normal localization of actin patches	0.866751	3.49E-21	up
PAS_chr1-1_0169	Protein required for the ubiquinone (Coenzyme Q) biosynthesis	0.651011	7.58E-14	up
PAS_chr1-1_0176	Putative FAD transporter	0.619806	1.12E-10	up
PAS_chr1-1_0180	GTPase; GTP-binding protein of the ARF family, component of COPII coat of vesicles	0.829514	1.19E-26	up
PAS_chr1-1_0183	60S ribosomal protein L1	1.330829	2.06E-49	up
PAS_chr1-1_0189	60S ribosomal protein L12	1.367465	6.46E-45	up
PAS_chr1-1_0191	Subunit 5 of the stator stalk of mitochondrial F1F0 ATP synthase	0.676772	5.18E-17	up
PAS_chr1-1_0198	Hypothetical protein	0.984007	1.64E-22	up
PAS_chr1-1_0200	Cytosolic aspartate aminotransferase; involved in nitrogen metabolism	0.844364	1.46E-22	up
PAS_chr1-1_0201	G protein beta subunit, forms a dimer with Ste18p to activate the mating signaling pathway	0.683762	1.76E-08	up
PAS_chr1-1_0216	60S ribosomal protein L8	1.095821	4.56E-34	up
PAS_chr1-1_0219	60S ribosomal protein L2	1.333702	1.79E-50	up
PAS_chr1-1_0221	Low affinity methionine permease, similar to Mup1p	0.832369	6.02E-21	up
PAS_chr1-1_0231	Putative protein of unknown function	0.706049	5.36E-10	up
PAS_chr1-1_0233	Mitochondrial NADP-specific isocitrate dehydrogenase, catalyzes the oxidation of isocitrate	0.770809	1.94E-19	up
PAS_chr1-1_0252	Hypothetical protein	0.661845	5.55E-13	up
PAS_chr1-1_0257	Hypothetical protein	0.997228	1.78E-30	up
PAS_chr1-1_0267	Peptidyl-prolyl cis-trans isomerase (cyclophilin) of the endoplasmic reticulum	1.007962	5.01E-29	up
PAS_chr1-1_0274	Catalytic subunit of the mitochondrial inner membrane peptidase complex	0.718464	1.48E-06	up
PAS_chr1-1_0292	Protein involved in rRNA processing	0.633472	1.84E-10	up
PAS_chr1-1_0293	Cell wall protein that functions in the transfer of chitin to beta(1-6)glucan	0.660287	8.66E-16	up
PAS_chr1-1_0305	Rho GDP dissociation inhibitor involved in the localization and regulation of Cdc42p	0.633709	2.00E-10	up
PAS_chr1-1_0309	Vacuolar ATPase V1 domain subunit A containing the catalytic nucleotide binding sites	0.746844	5.94E-17	up
PAS_chr1-1_0345	60S ribosomal protein L5	0.960136	2.93E-25	up
PAS_chr1-1_0347	Hypothetical protein	1.098655	0.020597	up
PAS_chr1-1_0371	Transcription factor (bHLH) involved in interorganelle communication	0.835919	2.00E-11	up
PAS_chr1-1_0401	Mitochondrial protein, putative inner membrane transporter	0.834015	4.85E-12	up
PAS_chr1-1_0410	Vacuolar ATPase assembly integral membrane protein VMA21	1.206226	2.93E-06	up
PAS_chr1-1_0417	Putative transmembrane protein involved in export of ammonia	0.88664	8.31E-25	up
PAS_chr1-1_0418	Acetate transporter required for normal sporulation	1.156211	3.99E-23	up
PAS_chr1-1_0432	Acetohydroxyacid reductoisomerase	0.796501	7.57E-21	up
PAS_chr1-1_0433	Mitochondrial peroxiredoxin (1-Cys Prx) with thioredoxin peroxidase activity	0.880485	4.43E-28	up
PAS_chr1-1_0439	40S ribosomal protein S8	0.592496	1.61E-16	up
PAS_chr1-1_0459	Dolichol-phosphate mannosyltransferase	0.764069	3.65E-21	up
PAS_chr1-1_0461	hypothetical protein	0.706605	3.11E-13	up
PAS_chr1-1_0475	hypothetical protein	0.99958	5.30E-31	up
PAS_chr1-1_0479	hypothetical protein	0.645947	4.35E-16	up
PAS_chr1-1_0482	hypothetical protein	0.85606	2.07E-18	up
PAS_chr1-1_0484	hypothetical protein	1.399526	2.48E-38	up
PAS_chr1-1_0496	hypothetical protein	0.801687	7.86E-10	up
PAS_chr1-3_0004	Hexose transporter with moderate affinity for glucose	0.618043	1.35E-06	up
PAS_chr1-3_0011	Putative transporter, member of the sugar porter family	0.968964	6.82E-25	up
PAS_chr1-3_0016	Multifunctional enzyme of the peroxisomal fatty acid beta-oxidation pathway	0.790733	8.47E-10	up
PAS_chr1-3_0024	Succinate semialdehyde dehydrogenase	0.957622	2.31E-28	up

PAS_chr1-3_0027	Protein of unknown function, expression is regulated by phosphate levels	1.220137	1.42E-32	up
PAS_chr1-3_0028	Cytoplasmic inorganic pyrophosphatase (PPase)	1.115389	8.23E-37	up
PAS_chr1-3_0034	60S ribosomal protein L4	1.159051	1.11E-35	up
PAS_chr1-3_0059	Uracil permease, localized to the plasma membrane	0.880994	2.36E-25	up
PAS_chr1-3_0068	60S acidic ribosomal protein P0	1.175524	6.50E-45	up
PAS_chr1-3_0070	Mitochondrial inorganic pyrophosphatase	0.718134	5.11E-17	up
PAS_chr1-3_0075	Hypothetical protein	1.038597	1.20E-18	up
PAS_chr1-3_0077	Nuclear protein of unknown function	0.866797	6.15E-21	up
PAS_chr1-3_0104	Aconitase, required for the tricarboxylic acid (TCA) cycle and also independently required for mitoc	0.964765	3.08E-28	up
PAS_chr1-3_0113	Small rho-like GTPase, essential for establishment and maintenance of cell polarity	0.833201	7.07E-15	up
PAS_chr1-3_0115	40S ribosomal protein S3	0.987901	5.52E-27	up
PAS_chr1-3_0116	Endoplasmic reticulum packaging chaperone	0.626392	4.57E-15	up
PAS_chr1-3_0117	Hypothetical protein	1.092754	2.81E-17	up
PAS_chr1-3_0120	Hypothetical protein	0.646502	0.001337	up
PAS_chr1-3_0141	Protein with a role in cellular adhesion and filamentous growth	0.59202	1.10E-07	up
PAS_chr1-3_0146	Hypothetical protein	0.665816	1.42E-06	up
PAS_chr1-3_0149	Transmembrane osmosensor	1.039675	5.10E-21	up
PAS_chr1-3_0153	Plasma membrane pyridoxine (vitamin B6) transporter	1.189058	5.49E-26	up
PAS_chr1-3_0162	Highly conserved, iron-sulfur cluster binding protein localized in the cytoplasm	0.775709	8.19E-11	up
PAS_chr1-3_0163	Uridine diphosphate-N-acetylglucosamine (UDP-GlcNAc) transporter	0.918906	6.03E-13	up
PAS_chr1-3_0164	Putative protein of unknown function	0.73053	2.67E-07	up
PAS_chr1-3_0166	Hypothetical protein	0.716509	7.78E-17	up
PAS_chr1-3_0170	Zinc-finger DNA-binding protein	0.790579	1.77E-05	up
PAS_chr1-3_0172	Subunit beta1 of the nascent polypeptide-associated complex (NAC) involved in protein targeting	0.58545	1.66E-14	up
PAS_chr1-3_0186	Putative protein, predicted to be an alpha-isopropylmalate carrier	0.872878	1.15E-09	up
PAS_chr1-3_0191	GMP synthase, an enzyme that catalyzes the second step in the biosynthesis of GMP from IMP	1.190074	2.53E-34	up
PAS_chr1-3_0194	Heme A:farnesyltransferase	0.689446	3.49E-08	up
PAS_chr1-3_0202	Essential subunit of Sec61 complex (Sec61p, Sbh1p, and Sss1p)	0.854822	6.24E-21	up
PAS_chr1-3_0206	Permease of basic amino acids in the vacuolar membrane	0.599762	1.36E-07	up
PAS_chr1-3_0208	Mitochondrial peripheral inner membrane protein	0.636568	6.39E-09	up
PAS_chr1-3_0226	Beta-1,3-glucanosyltransferase, required for cell wall assembly	0.828314	8.18E-18	up
PAS_chr1-3_0227	Beta-1,3-glucanosyltransferase, required for cell wall assembly	0.817231	8.57E-21	up
PAS_chr1-3_0229	Cell wall protein with similarity to glucanases	1.195984	2.29E-36	up
PAS_chr1-3_0230	ER membrane protein that interacts with exocyst subunit Sec6p and with Yip3p	0.930316	1.49E-25	up
PAS_chr1-3_0249	Hypothetical protein	0.849597	2.02E-16	up
PAS_chr1-3_0250	Mitochondrial carrier protein involved in the accumulation of CoA in the mitochondrial matrix	0.80261	1.06E-15	up
PAS_chr1-3_0256	Hypothetical protein	1.078426	1.06E-18	up
PAS_chr1-3_0264	Cytoplasmic peptidyl-prolyl cis-trans isomerase (cyclophilin)	1.128444	5.80E-36	up
PAS_chr1-3_0276	Mucin family member	0.721486	5.49E-11	up
PAS_chr1-3_0300	60S ribosomal protein L23	0.610911	1.29E-15	up
PAS_chr1-3_0301	hypothetical protein	0.852991	1.31E-20	up
PAS_chr1-4_0027	Cofilin, promotes actin filament depolarization in a pH-dependent manner	0.820692	1.15E-27	up
PAS_chr1-4_0043	Co-chaperone that binds to and regulates Hsp90 family chaperones	0.636475	1.53E-14	up
PAS_chr1-4_0045	RNA binding protein with similarity to hnRNP-K that localizes to the cytoplasm and subtelomeric DNA	0.610794	1.51E-16	up
PAS_chr1-4_0049	Homoserine dehydrogenase (L-homoserine:NADP oxidoreductase), dimeric enzyme	0.755418	2.03E-18	up

PAS_chr1-4_0055	Clavaminate synthase	0.858522	4.92E-22	up
PAS_chr1-4_0057	Protein of unknown function, required for growth on glycerol as a carbon source	0.632689	1.92E-10	up
PAS_chr1-4_0063	G-protein beta subunit and guanine nucleotide dissociation inhibitor for Gpa2p	1.184743	2.82E-42	up
PAS_chr1-4_0086	Hypothetical protein	0.875806	2.76E-18	up
PAS_chr1-4_0116	Hypothetical protein	0.652145	4.30E-08	up
PAS_chr1-4_0126	Palmitoyltransferase for Vac8p, required for vacuolar membrane fusion	0.77641	3.58E-10	up
PAS_chr1-4_0150	Transketolase, similar to Tkl2p	0.876892	9.84E-21	up
PAS_chr1-4_0151	Protein phosphotyrosine phosphatase	0.702901	2.09E-12	up
PAS_chr1-4_0163	Putative protein of unknown function	1.089674	1.28E-24	up
PAS_chr1-4_0164	Protein of unknown function, has similarity to Pry1p and Pry3p	0.746361	1.57E-16	up
PAS_chr1-4_0167	Putative protein of unknown function	0.910796	3.63E-21	up
PAS_chr1-4_0168	hypothetical protein	0.685898	6.39E-14	up
PAS_chr1-4_0207	Hydroperoxide and superoxide-radical responsive glutathione-dependent oxidoreductase	0.735618	9.61E-16	up
PAS_chr1-4_0208	RNA helicase in the DEAH-box family involved in the second catalytic step of splicing, exhibits ATP-	0.891921	2.97E-22	up
PAS_chr1-4_0223	RNA binding protein that negatively regulates growth rate	0.730558	4.44E-13	up
PAS_chr1-4_0225	ER-derived vesicles protein ERV14	0.691388	7.21E-10	up
PAS_chr1-4_0242	Putative mannosidase, GPI-anchored membrane protein	0.955737	1.07E-27	up
PAS_chr1-4_0243	Threonine deaminase, catalyzes the first step in isoleucine biosynthesis	0.605219	6.92E-08	up
PAS_chr1-4_0246	Divalent metal ion transporter involved in manganese homeostasis	0.637679	5.98E-08	up
PAS_chr1-4_0248	Essential protein of the mitochondrial inner membrane, component of the mitochondrial import system	1.038436	2.78E-25	up
PAS_chr1-4_0251	Putative protein of unknown function	0.671754	0.000855	up
PAS_chr1-4_0253	ATP sulfurylase, catalyzes the primary step of intracellular sulfate activation	1.282791	1.10E-40	up
PAS_chr1-4_0257	Protein with similarity to mammalian monocarboxylate permeases	0.692937	4.33E-09	up
PAS_chr1-4_0260	Hypothetical protein	0.712549	3.73E-12	up
PAS_chr1-4_0261	Hypothetical protein	0.674785	0.001239	up
PAS_chr1-4_0264	Phosphoglucomutase	0.847256	6.90E-26	up
PAS_chr1-4_0270	Hypothetical protein	0.815299	7.58E-14	up
PAS_chr1-4_0276	WD-repeat protein involved in ribosome biogenesis	0.727534	3.79E-12	up
PAS_chr1-4_0290	Hypothetical protein	0.638094	6.66E-08	up
PAS_chr1-4_0292	3-phosphoglycerate kinase	0.798834	2.60E-26	up
PAS_chr1-4_0297	Suppressor protein STM1	0.854605	3.02E-26	up
PAS_chr1-4_0299	Mitochondrial external NADH dehydrogenase, a type II NAD(P)H:quinone oxidoreductase	1.09372	4.71E-36	up
PAS_chr1-4_0301	Hypothetical protein	0.699413	2.04E-06	up
PAS_chr1-4_0304	Acetyl-CoA C-acetyltransferase (acetoacetyl-CoA thiolase), cytosolic enzyme	0.839695	8.54E-23	up
PAS_chr1-4_0340	ADP-ribosylation factor (ARF) GTPase activating protein (GAP) effector	0.645011	2.09E-08	up
PAS_chr1-4_0350	Putative protein of unknown function	0.698862	2.99E-09	up
PAS_chr1-4_0352	60S ribosomal protein L18	1.00642	9.49E-30	up
PAS_chr1-4_0353	40S ribosomal protein S19	1.210182	4.56E-43	up
PAS_chr1-4_0369	Hypothetical protein	0.986551	0.003581	up
PAS_chr1-4_0370	hypothetical protein	0.626954	1.64E-08	up
PAS_chr1-4_0371	Hypothetical protein	1.030226	9.05E-27	up
PAS_chr1-4_0374	Subunit of an adoMet-dependent tRNA methyltransferase (MTase) complex (Trm11p-Trm112p)	1.079046	0.000495	up
PAS_chr1-4_0394	Ammonium permease involved in regulation of pseudohyphal growth	1.260032	1.07E-31	up
PAS_chr1-4_0419	Component of the mitotic exit network	0.598661	4.83E-11	up
PAS_chr1-4_0421	Homocitrate synthase isozyme, catalyzes the	0.919227	7.49E-24	up

	condensation of acetyl-CoA and alpha-ketoglutarate			
PAS_chr1-4_0422	40S ribosomal protein S9	1.348526	9.15E-41	up
PAS_chr1-4_0426	Endo-beta-1,3-glucanase, major protein of the cell wall, involved in cell wall maintenance	1.099303	1.20E-34	up
PAS_chr1-4_0429	Isopentenyl diphosphate:dimethylallyl diphosphate isomerase (IPP isomerase)	0.629771	5.39E-08	up
PAS_chr1-4_0445	Delta subunit of the central stalk of mitochondrial F1F0 ATP synthase	1.204213	3.42E-32	up
PAS_chr1-4_0471	40S ribosomal protein S0	1.014367	1.04E-33	up
PAS_chr1-4_0481	Hypothetical protein	0.6607	7.13E-06	up
PAS_chr1-4_0487	Putative protein of unknown function	0.708566	1.63E-17	up
PAS_chr1-4_0489	Cystathionine gamma-lyase	0.794734	9.52E-19	up
PAS_chr1-4_0490	60S acidic ribosomal protein P2	0.992411	3.69E-25	up
PAS_chr1-4_0510	CFEM protein	0.620759	6.15E-15	up
PAS_chr1-4_0518	Hypothetical protein	0.688543	2.89E-08	up
PAS_chr1-4_0531	Hypothetical protein	1.279607	1.26E-28	up
PAS_chr1-4_0537	Protein of unknown function, similar to Listeria monocytogenes major sigma factor	1.003178	1.50E-28	up
PAS_chr1-4_0540	Hypothetical protein	0.726498	0.007869	up
PAS_chr1-4_0547	peroxiredoxin	0.824597	2.68E-22	up
PAS_chr1-4_0548	Vacuolar proteinase B (yscB), a serine protease of the subtilisin family	0.970816	1.00E-23	up
PAS_chr1-4_0552	Ammonium permease	0.716364	3.55E-13	up
PAS_chr1-4_0553	hypothetical protein	0.843769	1.58E-07	up
PAS_chr1-4_0557	mitochondrial 54S ribosomal protein YmL47	0.623873	1.02E-09	up
PAS_chr1-4_0569	Primary component of eisosomes	0.939944	5.67E-32	up
PAS_chr1-4_0576	Hypothetical protein	0.806466	5.72E-14	up
PAS_chr1-4_0577	Mitochondrial inner membrane insertase	0.95996	1.99E-19	up
PAS_chr1-4_0582	Hypothetical protein	0.801275	4.64E-06	up
PAS_chr1-4_0584	Lectin-like protein with similarity to Flo1p, thought to be expressed and involved in flocculation	1.330701	1.45E-57	up
PAS_chr1-4_0586	Hypothetical protein	0.761944	8.58E-29	up
PAS_chr1-4_0587	Hypothetical protein	0.864883	7.05E-17	up
PAS_chr1-4_0588	Hypothetical protein	1.021931	1.32E-32	up
PAS_chr1-4_0589	40S ribosomal protein S2	1.246937	7.25E-49	up
PAS_chr1-4_0593	E1 beta subunit of the pyruvate dehydrogenase (PDH) complex	1.221457	4.68E-33	up
PAS_chr1-4_0602	N(6)-adenine-specific DNA methyltransferase	1.052688	1.61E-18	up
PAS_chr1-4_0604	C-3 sterol dehydrogenase	1.288868	2.37E-38	up
PAS_chr1-4_0611	Vacuolar aminopeptidase Y, processed to mature form by Prb1p	1.049777	1.17E-34	up
PAS_chr1-4_0612	Hypothetical protein	1.105411	9.91E-30	up
PAS_chr1-4_0615	mitochondrial phosphate carrier protein	1.202654	1.67E-44	up
PAS_chr1-4_0617	Phosphatidylinositol (PI) phosphatase	0.688969	2.97E-14	up
PAS_chr1-4_0629	Subunit of the Ssh1 translocon complex	1.041198	3.05E-23	up
PAS_chr1-4_0669	hypothetical protein	0.725946	2.81E-05	up
PAS_chr1-4_0672	hypothetical protein	0.986176	1.61E-06	up
PAS_chr1-4_0676	hypothetical protein	1.313537	4.70E-19	up
PAS_chr1-4_0677	hypothetical protein	0.726256	1.57E-07	up
PAS_chr1-4_0685	Dolichyl-diphosphooligosaccharide--protein glycosyltransferase subunit STT3	0.841677	1.40E-17	up
PAS_chr1-4_0686	Small nuclear ribonucleoprotein-associated protein	0.862555	5.16E-06	up
PAS_chr1-4_0690	hypothetical protein	0.698631	1.57E-05	up
PAS_chr1-4_0703	hypothetical protein	1.056813	6.44E-30	up
PAS_chr2-1_0021	Hypothetical protein	1.07149	6.80E-28	up
PAS_chr2-1_0022	60S ribosomal protein L7	0.680059	3.31E-16	up

PAS_chr2-1_0032	Putative protein of unknown function with similarity to glutamine amidotransferase proteins	0.601403	4.53E-07	up
PAS_chr2-1_0037	Nitrilase, member of the nitrilase branch of the nitrilase superfamily	0.90322	1.50E-14	up
PAS_chr2-1_0055	Plasma membrane transporter for both urea and polyamines, expression is highly sensitive to nitrogen	0.827653	7.41E-15	up
PAS_chr2-1_0072	Delta(9) fatty acid desaturase	0.845317	4.99E-29	up
PAS_chr2-1_0086	60S ribosomal protein L24	0.675782	9.84E-20	up
PAS_chr2-1_0087	60S ribosomal protein L30	1.01438	9.36E-27	up
PAS_chr2-1_0088	Hypothetical protein	0.792586	2.56E-17	up
PAS_chr2-1_0092	Pho85 cyclin of the Pcl1,2-like subfamily, involved in entry into the mitotic cell cycle and regulat	1.318779	1.68E-06	up
PAS_chr2-1_0105	Shuttling pre-60S factor	0.696182	5.40E-11	up
PAS_chr2-1_0111	Mitochondrial adenylate kinase, catalyzes the reversible synthesis of GTP and AMP from GDP and ADP	0.608993	6.97E-13	up
PAS_chr2-1_0112	Small rho-like GTPase, essential for establishment and maintenance of cell polarity	0.853574	1.49E-22	up
PAS_chr2-1_0117	GTPase activating protein (GAP) for Rho1p, involved in signaling to the actin cytoskeleton, null mut	0.64547	1.90E-09	up
PAS_chr2-1_0120	Subunit of mitochondrial NAD(+)-dependent isocitrate dehydrogenase, which catalyzes the oxidation of	0.840664	3.40E-22	up
PAS_chr2-1_0142	Subunit (17 kDa) of TFIID and SAGA complexes, involved in RNA polymerase II transcription initiation	0.824532	1.10E-15	up
PAS_chr2-1_0143	Conserved protein of the mitochondrial matrix, performs a scaffolding function during assembly of ir	0.906086	5.94E-22	up
PAS_chr2-1_0167	Putative metalloprotease, similar to O-sialoglycoprotein metallopeptidase from P. haemolytica	0.711938	1.34E-08	up
PAS_chr2-1_0183	Acyl-protein thioesterase responsible for depalmitoylation of Gpa1p	0.647586	8.83E-11	up
PAS_chr2-1_0191	3-hydroxy-3-methylglutaryl-CoA (HMG-CoA) synthase, catalyzes the formation of HMG-CoA from acetyl-Co	0.622884	6.43E-13	up
PAS_chr2-1_0197	D-Arabinono-1,4-lactone oxidase, catalyzes the final step in biosynthesis of D-erythroascorbic acid	0.629359	6.99E-11	up
PAS_chr2-1_0198	Fe(II)-dependent sulfonate/alpha-ketoglutarate dioxygenase, involved in sulfonate catabolism for use	1.096746	4.36E-19	up
PAS_chr2-1_0212	Protein O-mannosyltransferase, transfers mannose residues from dolichyl phosphate-D-mannose to prote	0.633852	2.24E-12	up
PAS_chr2-1_0218	Hypothetical protein	0.822813	2.48E-24	up
PAS_chr2-1_0233	Cytoplasmic RNA-binding protein, contains an RNA recognition motif (RRM)	1.091501	4.12E-16	up
PAS_chr2-1_0235	Na+/Pi cotransporter, active in early growth phase	1.153777	1.02E-26	up
PAS_chr2-1_0238	Mitochondrial malate dehydrogenase, catalyzes interconversion of malate and oxaloacetate	0.802303	3.71E-25	up
PAS_chr2-1_0253	Hypothetical protein	0.821071	7.19E-25	up
PAS_chr2-1_0256	Protein O-mannosyltransferase, transfers mannose residues from dolichyl phosphate-D-mannose to prote	0.680932	6.75E-14	up
PAS_chr2-1_0257	Nucleoside transporter with broad nucleoside selectivity	0.920522	9.87E-17	up
PAS_chr2-1_0270	Plasma membrane protein with roles in the uptake of protoprophyrin IX and the efflux of heme	0.861018	8.75E-06	up
PAS_chr2-1_0272	Hypothetical protein	0.666323	9.91E-09	up
PAS_chr2-1_0273	Protein involved in bud-site selection	0.91474	2.73E-17	up
PAS_chr2-1_0279	Triacylglycerol lipase of the lipid particle, responsible for all the TAG lipase activity of the lip	0.671058	2.74E-13	up
PAS_chr2-1_0293	Hypothetical protein	0.774788	1.06E-15	up
PAS_chr2-1_0309	Protein with similarity to mammalian monocarboxylate permeases	0.621909	8.51E-11	up
PAS_chr2-1_0313	Bifunctional enzyme with alcohol dehydrogenase and glutathione-dependent formaldehyde dehydrogenase	0.883244	9.14E-31	up
PAS_chr2-1_0314	Protein that binds tRNA and methionyl-and glutamyl-tRNA synthetases (Mes1p and Gus1p)	0.746363	1.65E-15	up
PAS_chr2-1_0333	Zeta-crystallin homolog, found in the cytoplasm and nucleus	1.09607	8.64E-30	up
PAS_chr2-1_0337	Hypothetical protein	0.848219	0.002951	up
PAS_chr2-1_0341	Hypothetical protein	0.728962	2.75E-15	up
PAS_chr2-1_0350	Protein that forms a complex with Spt5p and mediates both activation and inhibition of transcription	0.911415	9.39E-11	up
PAS_chr2-1_0351	High affinity methionine permease	1.342169	1.90E-39	up

PAS_chr2-1_0358	Cystathionine beta-lyase, converts cystathionine into homocysteine	0.720586	3.03E-16	up
PAS_chr2-1_0362	40S ribosomal protein S26	1.053235	6.80E-24	up
PAS_chr2-1_0363	Subunit VIa of cytochrome c oxidase, which is the terminal member of the mitochondrial inner membran	0.842405	1.61E-24	up
PAS_chr2-1_0365	Putative protein of unknown function	1.09476	1.53E-31	up
PAS_chr2-1_0376	Putative GTPase, member of the Obg family	1.192726	2.60E-42	up
PAS_chr2-1_0383	L-homoserine-O-acetyltransferase, catalyzes the conversion of homoserine to O-acetyl homoserine	0.618935	5.48E-11	up
PAS_chr2-1_0415	Alpha-isopropylmalate synthase (2-isopropylmalate synthase)	0.621278	1.21E-17	up
PAS_chr2-1_0428	One of two nearly identical (see HTB1) histone H2B subtypes	0.946519	7.15E-22	up
PAS_chr2-1_0437	Glyceraldehyde-3-phosphate dehydrogenase, isozyme 3, involved in glycolysis and gluconeogenesis	1.016714	9.70E-38	up
PAS_chr2-1_0449	GTP binding protein (mammalian Ranp homolog)	0.802649	3.86E-22	up
PAS_chr2-1_0450	Component of the U1 snRNP complex required for pre-mRNA splicing	0.808233	7.81E-15	up
PAS_chr2-1_0451	Hypothetical protein	1.498919	6.26E-29	up
PAS_chr2-1_0472	Mitochondrial alcohol dehydrogenase isozyme III	1.48904	2.16E-54	up
PAS_chr2-1_0474	Hypothetical protein	0.648968	4.71E-05	up
PAS_chr2-1_0481	40S ribosomal protein S14	1.450698	3.85E-51	up
PAS_chr2-1_0482	40S ribosomal protein S22	0.761867	1.89E-21	up
PAS_chr2-1_0502	Thiol-specific peroxiredoxin, reduces hydroperoxides to protect against oxidative damage	1.215982	6.53E-36	up
PAS_chr2-1_0504	Peroxisomal membrane protein	0.75754	7.32E-23	up
PAS_chr2-1_0505	Subunit of the GINS complex (Sld5p, Psf1p, Psf2p, Psf3p)	0.722682	2.33E-13	up
PAS_chr2-1_0522	Protein that associates with ribosomes	1.343337	4.43E-49	up
PAS_chr2-1_0526	Putative protein of unknown function	0.794736	7.07E-14	up
PAS_chr2-1_0536	Hypothetical protein	1.194894	1.10E-32	up
PAS_chr2-1_0538	Membrane protein that interacts with Yip1p to mediate membrane traffic	0.725957	2.46E-18	up
PAS_chr2-1_0539	Hypothetical protein	1.185769	2.89E-45	up
PAS_chr2-1_0542	Putative benzil reductase	1.042484	1.21E-33	up
PAS_chr2-1_0553	Deoxyhypusine hydroxylase, a HEAT-repeat containing metalloenzyme that catalyses hypusine formation	1.032113	6.52E-21	up
PAS_chr2-1_0555	Subunit of the cytosolic chaperonin Cct ring complex, related to Tcp1p	0.636402	7.02E-14	up
PAS_chr2-1_0578	Hypothetical protein	0.720057	1.94E-09	up
PAS_chr2-1_0593	Dihydroorotase, catalyzes the third enzymatic step in the de novo biosynthesis of pyrimidines	1.022646	2.79E-16	up
PAS_chr2-1_0621	mitochondrial 54S ribosomal protein YmL27	0.695848	1.24E-06	up
PAS_chr2-1_0633	Integral membrane component of endoplasmic reticulum-derived COPII-coated vesicles	0.587337	1.24E-08	up
PAS_chr2-1_0634	40S ribosomal protein S27	0.657475	3.18E-11	up
PAS_chr2-1_0637	Bifunctional chorismate synthase and flavin reductase	0.712385	7.12E-16	up
PAS_chr2-1_0648	Protein component of the H/ACA snoRNP pseudouridylase complex	0.829407	3.64E-19	up
PAS_chr2-1_0657	3-phosphoglycerate dehydrogenase, catalyzes the first step in serine and glycine biosynthesis	0.70152	2.87E-13	up
PAS_chr2-1_0658	60S ribosomal protein L15	0.893397	7.45E-21	up
PAS_chr2-1_0676	Hypothetical protein	0.704112	1.97E-07	up
PAS_chr2-1_0679	Tryptophan synthase involved in tryptophan biosynthesis	0.662168	1.38E-13	up
PAS_chr2-1_0687	Vacuolar ATPase V0 domain subunit c', involved in proton transport activity	0.620239	2.33E-10	up
PAS_chr2-1_0692	Protein with a role in UDP-galactose transport to the Golgi lumen	0.658642	0.000282	up
PAS_chr2-1_0719	Putative transporter, member of the SLC10 carrier family	0.716265	8.18E-16	up
PAS_chr2-1_0728	60S ribosomal protein L28	1.061395	3.68E-35	up
PAS_chr2-1_0731	Protein that binds tRNA and methionyl-and glutamyl-tRNA synthetases (Mes1p and Gus1p)	0.807816	7.06E-20	up
PAS_chr2-1_0758	Calmodulin	0.671265	9.24E-16	up

PAS_chr2-1_0806	hypothetical protein	0.770887	6.30E-14	up
PAS_chr2-1_0812	hypothetical protein	1.336759	2.34E-39	up
PAS_chr2-1_0815	hypothetical protein	0.832042	1.43E-19	up
PAS_chr2-1_0853	hypothetical protein	1.263009	5.35E-43	up
PAS_chr2-1_0858	hypothetical protein	0.900119	1.45E-12	up
PAS_chr2-1_0860	hypothetical protein	0.592121	0.000701	up
PAS_chr2-1_0864	hypothetical protein	1.216584	3.96E-29	up
PAS_chr2-1_0867	hypothetical protein	0.721357	6.66E-16	up
PAS_chr2-1_0876	hypothetical protein	1.199042	1.34E-38	up
PAS_chr2-1_0877	hypothetical protein	0.79158	2.54E-09	up
PAS_chr2-1_0887	hypothetical protein	0.595743	1.83E-14	up
PAS_chr2-2_0019	Peroxisomal 2,4-dienoyl-CoA reductase, auxiliary enzyme of fatty acid beta-oxidation	0.796436	4.14E-19	up
PAS_chr2-2_0034	Alkaline phosphatase specific for p-nitrophenyl phosphate	1.223096	1.24E-40	up
PAS_chr2-2_0042	Specificity factor required for Rsp5p-dependent ubiquitination	0.690733	3.38E-07	up
PAS_chr2-2_0053	Phosphomannomutase, involved in synthesis of GDP-mannose and dolichol-phosphate-mannose	0.686553	2.44E-20	up
PAS_chr2-2_0054	60S ribosomal protein L10	1.225521	1.89E-41	up
PAS_chr2-2_0058	Eukaryotic initiation factor (eIF) 2A	0.836025	2.15E-14	up
PAS_chr2-2_0059	Nucleoside diphosphate kinase	1.23065	3.49E-50	up
PAS_chr2-2_0062	Putative protein of unknown function	0.920119	4.93E-25	up
PAS_chr2-2_0063	NADPH-dependent 1-acyl dihydroxyacetone phosphate reductase	0.758292	3.06E-19	up
PAS_chr2-2_0064	Protein of the SUN family (Sim1p, Uth1p, Nca3p, Sun4p) that may participate in DNA replication	0.928215	3.28E-23	up
PAS_chr2-2_0084	Repressible alkaline phosphatase, a glycoprotein localized to the vacuole	0.646329	9.57E-13	up
PAS_chr2-2_0087	ADP-ribosylation factor, GTPase of the Ras superfamily	0.734212	6.05E-21	up
PAS_chr2-2_0093	Putative protein of unknown function	0.662959	2.01E-12	up
PAS_chr2-2_0102	Essential constituent of the mitochondrial inner membrane presequence translocase	0.839809	1.28E-11	up
PAS_chr2-2_0109	60S ribosomal protein L17	1.479479	1.06E-48	up
PAS_chr2-2_0111	NAD-dependent glycerol-3-phosphate dehydrogenase, key enzyme of glycerol synthesis	0.711548	1.35E-17	up
PAS_chr2-2_0113	Inositol 1-phosphate synthase	0.82252	0.028708	up
PAS_chr2-2_0115	Boron efflux transporter of the plasma membrane	0.695423	3.65E-10	up
PAS_chr2-2_0131	Catalase A, breaks down hydrogen peroxide in the peroxisomal matrix formed by acyl-CoA oxidase (Pox1	0.757797	6.78E-22	up
PAS_chr2-2_0133	Hypothetical protein	0.606415	4.66E-05	up
PAS_chr2-2_0145	Isozyme of methylenetetrahydrofolate reductase	0.594166	1.86E-10	up
PAS_chr2-2_0147	Hypothetical protein	0.605392	4.12E-13	up
PAS_chr2-2_0148	Cell wall protein that contains a putative GPI-attachment site	1.398167	1.21E-43	up
PAS_chr2-2_0156	Hypothetical protein	0.587983	1.71E-07	up
PAS_chr2-2_0160	Vacuolar amino acid transporter, exports aspartate and glutamate from the vacuole	0.757593	1.01E-14	up
PAS_chr2-2_0161	Putative protein of unknown function	0.967533	2.02E-14	up
PAS_chr2-2_0165	Beta subunit of the F1 sector of mitochondrial F1F0 ATP synthase	1.375356	2.74E-45	up
PAS_chr2-2_0168	Homo-isocitrate dehydrogenase, an NAD-linked mitochondrial enzyme	0.906166	3.20E-23	up
PAS_chr2-2_0180	One of 10 subunits of the transport protein particle (TRAPP) complex of the cis-Golgi	0.997646	5.58E-20	up
PAS_chr2-2_0198	Hypothetical protein	0.825656	1.29E-19	up
PAS_chr2-2_0206	Hypothetical protein	0.594586	6.27E-13	up
PAS_chr2-2_0214	Protein of unknown function involved in rRNA and ribosome biosynthesis	0.640799	3.20E-09	up
PAS_chr2-2_0229	60S ribosomal protein L6	0.790501	2.55E-21	up
PAS_chr2-2_0236	Hypothetical protein	0.797623	1.77E-24	up
PAS_chr2-2_0238	Threonyl-tRNA synthetase, essential cytoplasmic	0.636694	8.44E-10	up

	protein			
PAS_chr2-2_0256	Hypothetical protein	0.591955	7.58E-14	up
PAS_chr2-2_0257	40S ribosomal protein S20	1.179945	2.37E-38	up
PAS_chr2-2_0258	Hypothetical protein	0.859118	3.19E-19	up
PAS_chr2-2_0265	Hypothetical protein	0.605288	5.86E-08	up
PAS_chr2-2_0266	Subunit VIII of cytochrome c oxidase	0.872052	5.16E-14	up
PAS_chr2-2_0268	Hypothetical protein	0.620535	0.000269	up
PAS_chr2-2_0271	GPI-anchored cell wall protein of unknown function	0.774901	2.12E-21	up
PAS_chr2-2_0276	Plasma membrane sulfite pump involved in sulfite metabolism	0.763551	2.68E-19	up
PAS_chr2-2_0277	Protein of unknown function, localized to the mitochondrial outer membrane	0.70689	1.34E-08	up
PAS_chr2-2_0278	Protein with an apparent role in acetylation of N-terminal methionine residues	0.627291	9.08E-11	up
PAS_chr2-2_0280	Mitochondrial glycosylase/lyase	0.617057	5.06E-10	up
PAS_chr2-2_0291	Essential protein of the mitochondrial intermembrane space, forms a complex with Tim9p	0.892086	1.92E-16	up
PAS_chr2-2_0293	Cytochrome c heme lyase (holocytochrome c synthase), attaches heme to apo-cytochrome c	0.880144	1.68E-26	up
PAS_chr2-2_0294	E1 alpha subunit of the pyruvate dehydrogenase (PDH) complex	0.970961	1.19E-30	up
PAS_chr2-2_0298	Cyclin-dependent protein kinase regulatory subunit and adaptor	0.735226	1.11E-07	up
PAS_chr2-2_0300	Hypothetical protein	0.648363	3.08E-10	up
PAS_chr2-2_0301	Protein with seven cysteine-rich CCHC zinc-finger motifs, similar to human CNBP	0.992702	2.37E-28	up
PAS_chr2-2_0303	Putative flavin-dependent monooxygenase, involved in ubiquinone (Coenzyme Q) biosynthesis	0.846978	4.04E-24	up
PAS_chr2-2_0326	40S ribosomal protein S25	0.944334	1.19E-26	up
PAS_chr2-2_0329	Adenylosuccinate lyase, catalyzes two steps in the 'de novo' purine nucleotide biosynthetic pathway	0.683582	1.11E-19	up
PAS_chr2-2_0330	Phosphatidylinositol:ceramide phosphoinositol transferase (IPC synthase)	0.841319	7.46E-14	up
PAS_chr2-2_0331	Hypothetical protein	0.669982	2.84E-08	up
PAS_chr2-2_0334	Protein with similarity to mammalian electron transfer flavoprotein complex subunit ETF-alpha	0.959394	1.02E-25	up
PAS_chr2-2_0337	Transaldolase, enzyme in the non-oxidative pentose phosphate pathway	0.628315	1.10E-14	up
PAS_chr2-2_0338	Transaldolase, enzyme in the non-oxidative pentose phosphate pathway	0.748745	2.76E-17	up
PAS_chr2-2_0355	Mitochondrial inner membrane carnitine transporter	0.721926	9.60E-12	up
PAS_chr2-2_0391	Ammonium permease	1.200828	1.49E-39	up
PAS_chr2-2_0392	Mitochondrial porin (voltage-dependent anion channel), outer membrane protein	0.93167	1.98E-29	up
PAS_chr2-2_0394	Hypothetical protein	0.618219	5.93E-15	up
PAS_chr2-2_0402	Subunit of the cytosolic chaperonin Cct ring complex, related to Tcp1p	0.823896	4.45E-20	up
PAS_chr2-2_0406	Hypothetical protein	1.459812	9.82E-32	up
PAS_chr2-2_0430	Subunit 2 of the ubiquinol cytochrome-c reductase complex	0.859446	1.33E-22	up
PAS_chr2-2_0460	hypothetical protein	0.935514	1.25E-17	up
PAS_chr2-2_0462	hypothetical protein	0.842356	9.90E-22	up
PAS_chr2-2_0463	hypothetical protein	1.252464	1.08E-27	up
PAS_chr2-2_0482	hypothetical protein	0.913843	0.002622	up
PAS_chr2-2_0483	hypothetical protein	0.624043	0.030333	up
PAS_chr2-2_0485	hypothetical protein	0.87677	5.17E-18	up
PAS_chr2-2_0488	hypothetical protein	0.897418	5.39E-19	up
PAS_chr2-2_0489	hypothetical protein	0.893015	1.98E-29	up
PAS_chr3_0006	NADPH-dependent medium chain alcohol dehydrogenase	0.587603	8.41E-14	up
PAS_chr3_0015	Hypothetical protein	0.977252	4.46E-24	up
PAS_chr3_0016	Hypothetical protein	1.114461	3.44E-19	up
PAS_chr3_0023	Putative transporter, member of the sugar porter family	0.656843	1.22E-10	up

PAS_chr3_0025	Hypothetical protein	0.971593	0.047031	up
PAS_chr3_0030	Hypothetical protein	0.812063	1.77E-21	up
PAS_chr3_0036	S-adenosylmethionine transporter of the mitochondrial inner membrane, member of the mitochondrial ca	0.585814	0.003858	up
PAS_chr3_0039	Beta-isopropylmalate dehydrogenase (IMDH), catalyzes the third step in the leucine biosynthesis path	0.981158	1.46E-24	up
PAS_chr3_0040	Mitochondrial inner membrane transporter, exports 2-oxoadipate and 2-oxoglutarate from the mitochond	1.248574	6.55E-42	up
PAS_chr3_0053	C-4 methyl sterol oxidase, catalyzes the first of three steps required to remove two C-4 methyl grou	0.912293	1.58E-23	up
PAS_chr3_0059	Subunit f of the F0 sector of mitochondrial F1F0 ATP synthase, which is a large, evolutionarily cons	0.829748	2.14E-20	up
PAS_chr3_0064	Hypothetical protein	0.853744	0.013189	up
PAS_chr3_0066	Peptide methionine sulfoxide reductase, reverses the oxidation of methionine residues	0.646789	2.09E-15	up
PAS_chr3_0076	Protein of unknown function, has similarity to Pry1p and Pry3p and to the plant PR-1 class of pathog	0.88296	1.91E-23	up
PAS_chr3_0082	Enolase I, a phosphopyruvate hydratase that catalyzes the conversion of 2-phosphoglycerate to phosph	0.762712	6.24E-27	up
PAS_chr3_0086	Peroxisomal 2,4-dienoyl-CoA reductase, auxiliary enzyme of fatty acid beta-oxidation	0.643971	1.62E-14	up
PAS_chr3_0091	60S ribosomal protein L19	0.634284	1.76E-15	up
PAS_chr3_0099	Mitochondrial NAD+ transporter, involved in the transport of NAD+ into the mitochondria (see also YE	1.259747	1.12E-41	up
PAS_chr3_0119	Putative protein of unknown function	1.01702	8.58E-20	up
PAS_chr3_0123	Mitochondrial protein required for assembly of ubiquinol cytochrome-c reductase complex (cytochrome	1.021545	4.35E-10	up
PAS_chr3_0125	Putative protein of unknown function	0.857435	8.09E-15	up
PAS_chr3_0130	Farnesyl cysteine-carboxyl methyltransferase, mediates the carboxyl methylation step during C-termin	1.107316	5.84E-12	up
PAS_chr3_0138	Hypothetical protein	0.60062	4.44E-05	up
PAS_chr3_0149	Hypothetical protein	0.699814	2.94E-06	up
PAS_chr3_0150	GTP-binding protein of the rho subfamily of Ras-like proteins, involved in establishment of cell pol	1.002055	2.11E-28	up
PAS_chr3_0167	3,4-dihydroxy-2-butanone-4-phosphate synthase (DHBP synthase), required for riboflavin biosynthesis	1.401968	7.04E-39	up
PAS_chr3_0176	Mitochondrial ornithine acetyltransferase	0.876481	3.43E-20	up
PAS_chr3_0177	Mitochondrial inner membrane citrate transporter	0.69069	3.01E-14	up
PAS_chr3_0187	Putative protein of unknown function	0.735644	1.23E-10	up
PAS_chr3_0191	Hypothetical protein	0.671778	4.62E-10	up
PAS_chr3_0196	Hypothetical protein	1.227204	0.005174	up
PAS_chr3_0208	Hypothetical protein	0.806097	1.55E-16	up
PAS_chr3_0222	3-hydroxyacyl-CoA dehydratase	0.820892	2.89E-18	up
PAS_chr3_0227	Primary component of eisosomes	0.890026	2.32E-24	up
PAS_chr3_0236	Elongase	1.095226	6.28E-29	up
PAS_chr3_0238	Subunit of the ARP2/3 complex	1.214854	8.50E-35	up
PAS_chr3_0255	Mitochondrial GTP/GDP transporter	0.587864	2.93E-09	up
PAS_chr3_0257	Adenylate kinase	1.128348	5.94E-30	up
PAS_chr3_0258	Hypothetical protein	0.605008	6.21E-09	up
PAS_chr3_0277	6-phosphogluconate dehydrogenase (decarboxylating)	0.915623	4.85E-32	up
PAS_chr3_0287	Integral membrane protein localized to mitochondria (untagged protein) and eisosomes, immobile patch	1.216663	3.16E-36	up
PAS_chr3_0290	Protein component of the small (40S) ribosomal subunit	0.60647	7.03E-15	up
PAS_chr3_0299	Aspartic protease, attached to the plasma membrane via a glycosylphosphatidylinositol (GPI) anchor	0.752956	2.51E-12	up
PAS_chr3_0332	Hypothetical protein	0.702571	1.03E-12	up
PAS_chr3_0335	60S ribosomal protein L43	0.821567	5.66E-30	up
PAS_chr3_0341	Hypothetical protein	0.697249	1.07E-08	up
PAS_chr3_0349	NADP(+)-dependent dehydrogenase	0.815279	1.39E-17	up
PAS_chr3_0356	Hypothetical protein	1.262142	2.64E-29	up
PAS_chr3_0384	Hypothetical protein	0.94671	1.99E-12	up

PAS_chr3_0388	Methionine aminopeptidase	0.645561	7.99E-12	up
PAS_chr3_0404	Putative protein of unknown function	1.026468	3.08E-34	up
PAS_chr3_0410	L-ornithine transaminase (OTAse)	0.850508	1.30E-23	up
PAS_chr3_0422	Hypothetical protein	0.873494	1.82E-22	up
PAS_chr3_0444	Hypothetical protein	1.001757	4.29E-29	up
PAS_chr3_0451	Mitochondrial outer membrane protein	0.918827	3.17E-16	up
PAS_chr3_0458	Essential component of the Arp2/3 complex	0.602712	1.57E-12	up
PAS_chr3_0460	Subunit b of the stator stalk of mitochondrial F1F0 ATP synthase	0.707686	8.18E-18	up
PAS_chr3_0482	Putative alanine transaminase (glutamic pyruvic transaminase)	0.737493	1.57E-13	up
PAS_chr3_0528	Saccharopine dehydrogenase (NADP+, L-glutamate-forming)	0.852849	4.93E-23	up
PAS_chr3_0547	Endoplasmic reticulum membrane protein	0.77151	8.42E-22	up
PAS_chr3_0551	Vacuolar membrane protein of unknown function	0.642942	5.37E-08	up
PAS_chr3_0558	Putative protein of unknown function	0.590589	0.000163	up
PAS_chr3_0562	Mitochondrial translation elongation factor Tu	1.20753	2.27E-36	up
PAS_chr3_0566	3-phosphoserine aminotransferase	0.632289	5.49E-15	up
PAS_chr3_0576	Alpha subunit of the F1 sector of mitochondrial F1F0 ATP synthase	1.046204	2.37E-32	up
PAS_chr3_0584	Component of the TOM (translocase of outer membrane) complex	0.738648	2.26E-09	up
PAS_chr3_0585	Hypothetical protein	1.135364	8.02E-36	up
PAS_chr3_0590	Homeodomain-containing transcriptional repressor of PTR2	0.676696	9.80E-08	up
PAS_chr3_0591	Cytosolic L-asparaginase	0.737054	6.67E-15	up
PAS_chr3_0595	Translation initiation factor eIF4A, identical to Tif1p	1.015357	8.62E-33	up
PAS_chr3_0596	40S ribosomal protein S21	0.955465	1.80E-27	up
PAS_chr3_0597	Long-chain base-1-phosphate phosphatase with specificity for dihydrosphingosine-1-phosphate	1.16072	8.41E-24	up
PAS_chr3_0598	Non-essential small GTPase of the Rho/Rac subfamily of Ras-like proteins	0.68693	1.21E-12	up
PAS_chr3_0615	Subunit Va of cytochrome c oxidase	0.854153	2.83E-23	up
PAS_chr3_0620	Alpha-1,6-mannosyltransferase involved in cell wall mannan biosynthesis	0.966333	5.50E-15	up
PAS_chr3_0623	Ornithine carbamoyltransferase (carbamoylphosphate:L-ornithine carbamoyltransferase)	0.844412	4.58E-18	up
PAS_chr3_0627	Protein that binds to cruciform DNA structures	0.620185	4.52E-16	up
PAS_chr3_0633	Putative serine type carboxypeptidase with a role in phytochelatin synthesis	0.752928	1.67E-19	up
PAS_chr3_0634	Tryptophan synthase involved in tryptophan biosynthesis	0.765965	1.35E-17	up
PAS_chr3_0640	Carboxypeptidase Y inhibitor, function requires acetylation by the NatB N-terminal acetyltransferase	0.903912	3.21E-22	up
PAS_chr3_0647	Fumarase, converts fumaric acid to L-malic acid in the TCA cycle	0.912239	1.44E-22	up
PAS_chr3_0648	Thiazole synthase, catalyzes formation of the thiazole moiety of thiamin pyrophosphate	0.685749	1.50E-17	up
PAS_chr3_0649	Transporter of thiamine or related compound	0.791224	1.78E-13	up
PAS_chr3_0662	Ferrioxamine B transporter	1.028	3.13E-29	up
PAS_chr3_0675	Asparagine synthetase, isozyme of Asn1p	0.685838	2.23E-12	up
PAS_chr3_0681	RNA exonuclease, required for U4 snRNA maturation	0.689442	6.66E-11	up
PAS_chr3_0697	Hypothetical protein	1.419277	5.84E-38	up
PAS_chr3_0709	Ubiquitin-conjugating enzyme most similar in sequence to Xenopus ubiquitin-conjugating enzyme E2-C	0.870754	5.06E-14	up
PAS_chr3_0714	Putative divalent metal ion transporter involved in iron homeostasis	0.602352	8.05E-12	up
PAS_chr3_0722	ubiquitin-40S ribosomal protein S31 fusion protein	0.90072	1.83E-29	up
PAS_chr3_0731	Cytoplasmic ATPase that is a ribosome-associated molecular chaperone	1.316478	1.28E-41	up
PAS_chr3_0735	Inositolphosphotransferase 1, involved in synthesis of mannose-(inositol-P)2-ceramide (M(IP)2C)	0.792795	3.95E-11	up
PAS_chr3_0743	Nit protein, one of two proteins in S. cerevisiae with similarity to the Nit domain	0.66426	1.87E-16	up

PAS_chr3_0744	Aldose reductase involved in methylglyoxal, d-xylose and arabinose metabolism	0.904051	1.89E-25	up
PAS_chr3_0746	Putative protein of unknown function	0.983458	4.07E-13	up
PAS_chr3_0762	40S ribosomal protein S5	1.14747	1.04E-37	up
PAS_chr3_0763	Spore-specific water channel that mediates the transport of water across cell membranes	1.322758	1.09E-39	up
PAS_chr3_0771	Hypothetical protein	0.587446	2.15E-09	up
PAS_chr3_0777	Putative protein of unknown function	0.598358	9.82E-06	up
PAS_chr3_0781	Glycogen synthase, similar to Gsy1p	0.587875	3.37E-14	up
PAS_chr3_0795	Protein involved in the organization of the actin cytoskeleton	0.722415	2.43E-19	up
PAS_chr3_0799	Large subunit of carbamoyl phosphate synthetase	0.706808	8.61E-17	up
PAS_chr3_0807	Mitochondrial intermembrane space cysteine motif protein	0.600004	4.90E-16	up
PAS_chr3_0808	Hypothetical protein	0.861411	3.68E-23	up
PAS_chr3_0809	Hypothetical protein	1.061103	2.69E-25	up
PAS_chr3_0819	Subunit g of the mitochondrial F1F0 ATP synthase	0.828112	7.13E-17	up
PAS_chr3_0826	Tetrameric phosphoglycerate mutase	1.221127	1.88E-52	up
PAS_chr3_0829	Hypothetical protein	0.656737	0.014476	up
PAS_chr3_0831	Alpha subunit of succinyl-CoA ligase	0.931795	8.08E-31	up
PAS_chr3_0832	Transketolase, similar to Tkl2p	1.120591	4.71E-36	up
PAS_chr3_0834	Transketolase, similar to Tkl2p	0.918945	2.87E-28	up
PAS_chr3_0839	Gamma subunit of the translation initiation factor eIF2	0.762739	5.55E-22	up
PAS_chr3_0842	Multifunctional protein with both hydroxymethylpyrimidine kinase and thiaminase activities	0.746299	1.79E-10	up
PAS_chr3_0843	Bifunctional enzyme	1.393798	2.28E-31	up
PAS_chr3_0867	Non-essential intracellular esterase that can function as an S-formylglutathione hydrolase	1.148165	3.59E-40	up
PAS_chr3_0868	Fructose-1,6-bisphosphatase, key regulatory enzyme in the gluconeogenesis pathway	1.055181	3.77E-42	up
PAS_chr3_0870	GDP-mannose pyrophosphorylase (mannose-1-phosphate guanyltransferase)	0.834489	5.91E-21	up
PAS_chr3_0875	Protein localized to COPII-coated vesicles, forms a complex with Erv46p	0.870593	1.75E-13	up
PAS_chr3_0890	S-adenosyl-L-homocysteine hydrolase	0.792796	4.84E-22	up
PAS_chr3_0899	Homoserine kinase, conserved protein required for threonine biosynthesis	1.014236	4.65E-40	up
PAS_chr3_0906	Mitochondrial peroxiredoxin (1-Cys Prx) with thioredoxin peroxidase activity	1.451619	8.57E-36	up
PAS_chr3_0909	Hypothetical protein	0.585894	0.035563	up
PAS_chr3_0919	Protein of unknown function	0.640831	6.71E-06	up
PAS_chr3_0932	NAD(+)-dependent formate dehydrogenase, may protect cells from exogenous formate	0.736431	2.17E-21	up
PAS_chr3_0946	mitochondrial 37S ribosomal protein RSM10	0.785393	5.79E-14	up
PAS_chr3_0951	Triose phosphate isomerase, abundant glycolytic enzyme	0.77508	3.41E-25	up
PAS_chr3_0952	Hypothetical protein	0.817287	6.83E-11	up
PAS_chr3_0954	Uroporphyrinogen decarboxylase	0.852973	3.18E-19	up
PAS_chr3_0960	Glycoprotein involved in cell wall beta-glucan assembly	0.878616	1.40E-16	up
PAS_chr3_0968	Transcription factor involved in cell-type-specific transcription and pheromone response	0.923194	4.79E-28	up
PAS_chr3_0970	Hypothetical protein	0.793652	1.08E-09	up
PAS_chr3_0971	Divalent metal ion transporter with a broad specificity for di-valent and tri-valent metals	0.916501	6.19E-28	up
PAS_chr3_0972	Cytoplasmic mRNA cap binding protein	0.758762	1.59E-23	up
PAS_chr3_0973	Hypothetical protein	0.590681	3.33E-12	up
PAS_chr3_0982	Ferric reductase and cupric reductase	0.84521	1.01E-18	up
PAS_chr3_0985	Putative protein of unknown function with similarity to acyl-carrier-protein reductases	0.887297	4.99E-15	up
PAS_chr3_0997	Cytochrome c1, component of the mitochondrial respiratory chain	0.924828	3.63E-28	up
PAS_chr3_0999	Glucosyl transferase, involved in N-linked	1.189757	1.73E-28	up

	glycosylation			
PAS_chr3_1004	Hypothetical protein	1.031233	1.74E-09	up
PAS_chr3_1015	Protein required for growth of cells lacking the mitochondrial genome	1.316731	1.68E-31	up
PAS_chr3_1028	S-(hydroxymethyl)glutathione dehydrogenase	0.838501	3.18E-27	up
PAS_chr3_1037	Lumazine synthase (6,7-dimethyl-8-ribityllumazine synthase, also known as DMRL synthase)	0.602926	1.49E-12	up
PAS_chr3_1040	Proteolipid subunit of the vacuolar H(+)-ATPase V0 sector	0.847267	2.82E-25	up
PAS_chr3_1041	small nucleolar ribonucleoprotein SNU13	0.612935	0.000184	up
PAS_chr3_1048	Translation initiation factor eIF-5A, promotes formation of the first peptide bond	0.878547	1.80E-30	up
PAS_chr3_1057	60S ribosomal protein L32	0.733086	2.80E-23	up
PAS_chr3_1058	Putative integral membrane protein	1.120289	1.58E-25	up
PAS_chr3_1071	Translation elongation factor EF-1 gamma	0.810834	3.20E-26	up
PAS_chr3_1087	Vacuolar aspartyl protease (proteinase A)	0.68714	1.22E-16	up
PAS_chr3_1089	Sporulation specific protein that localizes to the spore wall	0.954068	5.48E-22	up
PAS_chr3_1099	Glycerol proton symporter of the plasma membrane, subject to glucose-induced inactivation	1.016657	2.25E-25	up
PAS_chr3_1108	Plasma membrane protein involved in zinc metabolism and osmotin-induced apoptosis	0.597203	1.73E-07	up
PAS_chr3_1138	Adenine phosphoribosyltransferase, catalyzes the formation of AMP	1.053621	9.26E-28	up
PAS_chr3_1169	hypothetical protein	1.175288	4.35E-37	up
PAS_chr3_1199	hypothetical protein	0.720437	4.01E-19	up
PAS_chr3_1200	40S ribosomal protein S12	1.055874	7.91E-34	up
PAS_chr3_1215	hypothetical protein	1.184714	5.59E-23	up
PAS_chr3_1245	hypothetical protein	0.666505	7.59E-11	up
PAS_chr3_1256	hypothetical protein	1.007166	7.13E-17	up
PAS_chr4_0026	hypothetical protein	0.633705	6.28E-12	up
PAS_chr4_0038	Translational elongation factor 3, stimulates the binding of aminoacyl-tRNA (AA-tRNA) to ribosomes	0.830142	4.66E-18	up
PAS_chr4_0040	Protein of unknown function	0.662942	1.36E-16	up
PAS_chr4_0041	60S ribosomal protein L22	1.165788	1.47E-31	up
PAS_chr4_0043	Mitochondrial aldehyde dehydrogenase	1.560649	5.35E-40	up
PAS_chr4_0046	Mitochondrial outer membrane and cell wall localized SUN family member	1.196077	8.08E-35	up
PAS_chr4_0052	Hypothetical protein	0.883417	2.82E-22	up
PAS_chr4_0065	Protein involved in synthesis of the thiamine precursor hydroxymethylpyrimidine (HMP)	0.675766	0.000803	up
PAS_chr4_0075	Hypothetical protein	0.931877	1.96E-14	up
PAS_chr4_0102	elongation factor 1 gamma domain-containing protein	1.189995	9.69E-36	up
PAS_chr4_0104	Zinc-finger protein of unknown function	0.91239	0.000525	up
PAS_chr4_0107	60S ribosomal protein L2	1.095787	6.35E-33	up
PAS_chr4_0111	Low affinity methionine permease, similar to Mup1p	0.587521	6.71E-05	up
PAS_chr4_0112	Threonine aldolase	0.615323	3.99E-16	up
PAS_chr4_0116	Protein involved in N-glycosylation	1.19967	1.93E-18	up
PAS_chr4_0120	Hypothetical protein	0.883568	2.81E-24	up
PAS_chr4_0123	Receptor for alpha-factor pheromone	0.933331	1.97E-10	up
PAS_chr4_0131	60S ribosomal protein L6	1.317552	5.89E-44	up
PAS_chr4_0138	Small subunit of carbamoyl phosphate synthetase	0.753645	5.99E-20	up
PAS_chr4_0139	60S ribosomal protein L3	1.402596	1.74E-43	up
PAS_chr4_0140	ATP-dependent DEAD (Asp-Glu-Ala-Asp)-box RNA helicase	0.674604	3.03E-16	up
PAS_chr4_0157	Histone variant H2AZ, exchanged for histone H2A in nucleosomes by the SWR1 complex	0.875132	2.01E-28	up
PAS_chr4_0158	Tetradecameric mitochondrial chaperonin	0.640314	2.49E-17	up
PAS_chr4_0181	High-affinity copper transporter of the plasma membrane	0.6441	1.23E-10	up

PAS_chr4_0198	C-8 sterol isomerase	0.64971	1.24E-09	up
PAS_chr4_0201	Essential ATP-dependent RNA helicase of the DEAD-box protein family	0.601628	1.07E-11	up
PAS_chr4_0210	ADP/ATP carrier protein	1.212531	2.05E-42	up
PAS_chr4_0211	40S ribosomal protein S7	0.619272	2.71E-18	up
PAS_chr4_0212	Ribose-5-phosphate ketol-isomerase	0.721057	1.19E-20	up
PAS_chr4_0218	Mitochondrial type 2C protein phosphatase involved in regulation of pyruvate dehydrogenase activity	0.833772	5.46E-25	up
PAS_chr4_0222	Non-essential small GTPase of the Rho/Rac subfamily of Ras-like proteins	0.814195	2.50E-08	up
PAS_chr4_0224	Evolutionarily conserved subunit of the CCR4-NOT complex involved in controlling mRNA initiation	0.993661	8.33E-18	up
PAS_chr4_0246	40S ribosomal protein S4	1.106652	1.89E-41	up
PAS_chr4_0264	Dolichyl pyrophosphate (Dol-P-P) phosphatase with a luminally oriented active site in the ER	0.653219	6.71E-09	up
PAS_chr4_0280	Putative methylthio-ribulose-1-phosphate dehydratase	0.834888	3.76E-17	up
PAS_chr4_0283	Hypothetical protein	0.604866	3.60E-14	up
PAS_chr4_0284	Cytoplasmic thioredoxin isoenzyme of the thioredoxin system	0.696457	8.60E-14	up
PAS_chr4_0285	Phosphoserine phosphatase of the phosphoglycerate pathway, involved in serine and glycine biosynthes	0.869857	3.11E-25	up
PAS_chr4_0289	Hypothetical protein	0.8548	4.21E-07	up
PAS_chr4_0290	Vacuolar transporter chaperon (VTC) involved in distributing V-ATPase and other membrane proteins	0.691741	9.12E-07	up
PAS_chr4_0292	40S ribosomal protein S24	0.899591	1.13E-30	up
PAS_chr4_0305	O-glycosylated protein required for cell wall stability	0.825371	3.67E-16	up
PAS_chr4_0312	tRNA 2'-phosphotransferase	0.605775	1.88E-07	up
PAS_chr4_0330	Methionine and cysteine synthase (O-acetyl homoserine-O-acetyl serine sulfhydrylase)	1.376788	3.16E-50	up
PAS_chr4_0336	Putative dihydrokaempferol 4-reductase	0.75693	3.12E-15	up
PAS_chr4_0341	Hypothetical protein	0.652946	2.41E-11	up
PAS_chr4_0359	Hypothetical protein	1.219824	2.31E-24	up
PAS_chr4_0360	Hypothetical protein	1.199198	2.34E-29	up
PAS_chr4_0368	Microsomal beta-keto-reductase	0.928498	1.77E-19	up
PAS_chr4_0369	Subunit alpha of assimilatory sulfite reductase	0.950834	2.17E-21	up
PAS_chr4_0370	Protein that associates with the INO80 chromatin remodeling complex under low-salt conditions	0.687334	1.09E-08	up
PAS_chr4_0371	Hypothetical protein	0.732927	0.001278	up
PAS_chr4_0372	Putative protein of unknown function	0.646229	3.68E-12	up
PAS_chr4_0384	Hypothetical protein	0.720609	2.39E-16	up
PAS_chr4_0391	Component of the TOM (translocase of outer membrane) complex	0.958385	1.93E-21	up
PAS_chr4_0397	Protein of unknown function	0.791621	2.75E-05	up
PAS_chr4_0398	Delta 1-pyrroline-5-carboxylate reductase, catalyzes the last step in proline biosynthesis	1.091528	4.93E-23	up
PAS_chr4_0412	60S acidic ribosomal protein P1	1.46186	4.66E-49	up
PAS_chr4_0413	60S ribosomal protein L13	1.20846	2.78E-41	up
PAS_chr4_0414	40S ribosomal protein S16	0.754823	5.32E-24	up
PAS_chr4_0416	Alanine:glyoxylate aminotransferase (AGT), catalyzes the synthesis of glycine from glyoxylate	1.108035	4.85E-32	up
PAS_chr4_0418	Delta-aminolevulinate dehydratase, a homo-octameric enzyme	0.930272	1.16E-22	up
PAS_chr4_0427	Evolutionarily conserved protein with similarity to Orm2p	0.803861	7.99E-17	up
PAS_chr4_0456	40S ribosomal protein S13	0.931513	4.52E-30	up
PAS_chr4_0496	Peroxisomal ubiquitin conjugating enzyme	0.586612	1.29E-12	up
PAS_chr4_0504	Protein of unknown function	1.051155	6.08E-29	up
PAS_chr4_0514	Purine-cytosine permease	1.245106	5.66E-30	up
PAS_chr4_0524	40S ribosomal protein S1	1.378732	1.46E-41	up
PAS_chr4_0529	Hypothetical protein	1.140967	2.92E-20	up
PAS_chr4_0532	Permease of basic amino acids in the vacuolar membrane	0.615918	5.51E-10	up

PAS_chr4_0551	Protein of unknown function	1.028054	2.45E-22	up
PAS_chr4_0552	ATPase involved in protein folding and nuclear localization signal (NLS)-directed nuclear transport	0.654554	4.17E-18	up
PAS_chr4_0554	Hypothetical protein	0.678246	0.000254	up
PAS_chr4_0556	Hypothetical protein	0.619646	0.038167	up
PAS_chr4_0561	Mitochondrial intermembrane space protein, forms a complex with TIm8p	0.937794	1.51E-12	up
PAS_chr4_0562	Similar to globins and has a functional heme-binding domain	0.733033	1.25E-17	up
PAS_chr4_0571	Glycerol proton symporter of the plasma membrane, subject to glucose-induced inactivation	1.122012	1.68E-30	up
PAS_chr4_0572	Hypothetical protein	0.669737	5.14E-16	up
PAS_chr4_0578	Carbonic anhydrase	0.597933	9.25E-14	up
PAS_chr4_0580	Subunit of mitochondrial NAD(+)-dependent isocitrate dehydrogenase	0.654528	1.03E-18	up
PAS_chr4_0584	Aspartic protease, attached to the plasma membrane via a glycosylphosphatidylinositol (GPI) anchor	0.815905	4.22E-20	up
PAS_chr4_0587	Mitochondrial serine hydroxymethyltransferase	0.804076	5.42E-19	up
PAS_chr4_0588	Hypothetical protein	0.944311	2.84E-17	up
PAS_chr4_0589	Hypothetical protein	0.899287	1.96E-19	up
PAS_chr4_0592	Mitochondrial iron transporter of the mitochondrial carrier family (MCF)	0.894145	1.31E-17	up
PAS_chr4_0593	Glyoxylate reductase	0.593233	1.60E-12	up
PAS_chr4_0603	Acidic protein of the mitochondrial matrix involved in oxidative phosphorylation	0.983666	1.80E-30	up
PAS_chr4_0604	B-type cyclin involved in cell cycle progression	0.781978	2.85E-15	up
PAS_chr4_0613	Adenylosuccinate synthase	1.142608	2.91E-46	up
PAS_chr4_0624	Non-essential protein of unknown function required for transcriptional induction	0.856939	4.21E-24	up
PAS_chr4_0630	Hypothetical protein	0.905792	2.20E-10	up
PAS_chr4_0635	Hypothetical protein	0.660196	7.10E-10	up
PAS_chr4_0643	Hypothetical protein	0.826424	6.00E-22	up
PAS_chr4_0665	Gamma-glutamyl phosphate reductase, catalyzes the second step in proline biosynthesis	0.713704	1.15E-17	up
PAS_chr4_0668	Beta 5 subunit of the 20S proteasome, responsible for the chymotryptic activity of the proteasome	0.69469	2.75E-15	up
PAS_chr4_0672	Hypothetical protein	0.780112	9.57E-13	up
PAS_chr4_0677	Gamma-aminobutyrate (GABA) transaminase (4-aminobutyrate aminotransferase)	0.923762	5.70E-16	up
PAS_chr4_0682	Protein kinase that stabilizes several plasma membrane amino acid transporters	0.893978	6.69E-14	up
PAS_chr4_0686	Vacuolar carboxypeptidase yscS	1.196402	3.67E-34	up
PAS_chr4_0688	Mitochondrial succinate-fumarate transporter	0.73873	1.28E-10	up
PAS_chr4_0714	Mitochondrial serine protease	0.7583	2.56E-13	up
PAS_chr4_0726	Putative protein of unknown function	0.681046	4.54E-16	up
PAS_chr4_0733	Flavoprotein subunit of succinate dehydrogenase (Sdh1p, Sdh2p, Sdh3p, Sdh4p)	0.839747	8.57E-28	up
PAS_chr4_0737	Mitochondrial NADH-cytochrome b5 reductase, involved in ergosterol biosynthesis	1.002624	3.46E-27	up
PAS_chr4_0743	Hypothetical protein	0.947687	3.44E-19	up
PAS_chr4_0749	Golgi membrane protein involved in manganese homeostasis	0.813355	1.34E-06	up
PAS_chr4_0758	Integral membrane protein localized to late Golgi vesicles along with the v-SNARE Tlg2p	1.048335	1.76E-23	up
PAS_chr4_0780	Enoyl reductase that catalyzes the last step in each cycle of very long chain fatty acid elongation	0.646462	2.19E-10	up
PAS_chr4_0785	Glutamine synthetase (GS), synthesizes glutamine from glutamate and ammonia	1.194292	5.90E-45	up
PAS_chr4_0786	Cytosolic superoxide dismutase	0.876015	1.44E-30	up
PAS_chr4_0795	Homoaconitase, catalyzes the conversion of homocitrate to homoisocitrate, which is a step in the lys	0.916855	1.94E-23	up
PAS_chr4_0799	mitochondrial 54S ribosomal protein YmL7/YmL5	0.972767	1.71E-13	up
PAS_chr4_0807	Hypothetical protein	0.616628	0.000357	up
PAS_chr4_0811	Vacuolar membrane protein of unknown function involved in vacuolar protein sorting	0.672141	7.91E-12	up

PAS_chr4_0813	mitochondrial 37S ribosomal protein YmS18	0.984019	6.57E-21	up
PAS_chr4_0815	Mitochondrial malate dehydrogenase, catalyzes interconversion of malate and oxaloacetate	1.013822	7.81E-32	up
PAS_chr4_0821	Alcohol oxidase	1.186096	7.24E-35	up
PAS_chr4_0823	Polyamine oxidase, converts spermine to spermidine	0.911377	1.23E-23	up
PAS_chr4_0834	Hypothetical protein	0.703372	5.98E-06	up
PAS_chr4_0847	Self-glucosylating initiator of glycogen synthesis, also glucosylates n-dodecyl-beta-D-maltoside	0.814468	5.38E-21	up
PAS_chr4_0850	Hypothetical protein	0.804559	4.68E-18	up
PAS_chr4_0851	Hypothetical protein	0.816665	7.12E-16	up
PAS_chr4_0866	Mitochondrial membrane localized inositol phosphosphingolipid phospholipase C	0.600439	3.28E-09	up
PAS_chr4_0877	Mitochondrial dicarboxylate carrier, integral membrane protein	0.939385	6.92E-22	up
PAS_chr4_0884	hypothetical protein	0.858931	2.99E-12	up
PAS_chr4_0895	54S ribosomal protein L35, mitochondrial	0.710167	1.95E-14	up
PAS_chr4_0911	hypothetical protein	0.762581	8.45E-10	up
PAS_chr4_0914	hypothetical protein	0.755847	6.83E-11	up
PAS_chr4_0915	hypothetical protein	1.311754	3.77E-17	up
PAS_chr4_0924	hypothetical protein	0.675843	0.005973	up
PAS_chr4_0925	hypothetical protein	0.949742	2.04E-06	up
PAS_chr4_0926	hypothetical protein	0.70649	1.28E-13	up
PAS_chr4_0938	hypothetical protein	0.655012	4.24E-08	up
PAS_chr4_0940	hypothetical protein	0.61939	3.30E-13	up
PAS_chr4_0947	hypothetical protein	1.267107	2.54E-37	up
PAS_chr4_0948	hypothetical protein	1.008165	2.11E-24	up
PAS_chr4_0949	hypothetical protein	0.729193	0.000369	up
PAS_chr4_0972	hypothetical protein	1.055343	2.91E-22	up
PAS_chr4_0974	hypothetical protein	1.259507	3.06E-40	up
PAS_chr4_0978	hypothetical protein	0.901122	2.08E-21	up
PAS_chr4_0981	hypothetical protein	0.864567	4.41E-17	up
PAS_chr4_0982	60S acidic ribosomal protein P1	0.89199	4.77E-25	up
PAS_chr4_0988	hypothetical protein	0.827637	1.03E-14	up
PAS_chr4_0991	hypothetical protein	0.749494	2.90E-09	up
PAS_FragB_0027	AP-3 complex subunit delta	-0.60202	9.84E-09	down
PAS_FragB_0028	Hypothetical protein	-0.74244	5.45E-07	down
PAS_FragB_0037	54S ribosomal protein L24, mitochondrial	-0.76624	1.58E-08	down
PAS_FragB_0044	Transcriptional coactivator HFI1/ADA1	-0.86247	9.32E-11	down
PAS_FragB_0072	hypothetical protein	-0.69502	2.55E-05	down
PAS_FragD_0015	Mitochondrial RNA polymerase specificity factor	-0.58565	4.72E-06	down
PAS_c121_0019	Hypothetical protein	-0.59586	0.009671	down
PAS_c131_0006	Hypothetical protein	-0.9348	8.61E-17	down
PAS_chr1-1_0005	Inositol hexakisphosphate and inositol heptakisphosphate kinase	-0.65694	1.20E-08	down
PAS_chr1-1_0012	TFIID subunit (19 kDa), involved in RNA polymerase II transcription initiation	-0.70057	1.79E-05	down
PAS_chr1-1_0014	Lsm (Like Sm) protein	-0.78565	0.016685	down
PAS_chr1-1_0015	Putative endoribonuclease	-0.82702	1.20E-06	down
PAS_chr1-1_0018	Subunit of the heterodimeric FACT complex (Spt16p-Pob3p)	-0.86959	3.60E-16	down
PAS_chr1-1_0020	Actin-like protein	-0.61584	0.020447	down
PAS_chr1-1_0031	Cytoplasmic pre-60S factor	-0.86204	1.41E-12	down
PAS_chr1-1_0034	vacuolar sorting protein	-0.67776	0.0003	down
PAS_chr1-1_0043	Hypothetical protein	-1.07575	4.25E-20	down
PAS_chr1-1_0055	Protein that interacts with Cdc48p and Npl4p, involved in recognition of polyubiquitinated proteins	-1.01075	8.89E-12	down

PAS_chr1-1_0057	Putative protein serine/threonine kinase expressed at the end of meiosis	-0.73896	0.015512	down
PAS_chr1-1_0067	Essential N-acetylglucosamine-phosphate mutase	-0.69761	7.55E-10	down
PAS_chr1-1_0079	Non-ATPase regulatory subunit of the 26S proteasome	-0.98583	7.26E-31	down
PAS_chr1-1_0083	Kinesin-related motor protein involved in mitotic spindle positioning	-0.99742	7.48E-06	down
PAS_chr1-1_0088	Essential protein that binds ribosomal protein L11	-0.87982	8.10E-06	down
PAS_chr1-1_0108	Hypothetical protein	-0.74821	1.63E-06	down
PAS_chr1-1_0151	Protein that relieves transcriptional repression by binding to the Cyc8p-Tup1p corepressor	-0.98899	3.29E-09	down
PAS_chr1-1_0230	tRNA methyltransferase, methylates the N-1 position of guanosine in tRNAs	-0.64909	0.000467	down
PAS_chr1-1_0237	Nucleotide exchange factor for the endoplasmic reticulum (ER) lumenal Hsp70 chaperone Kar2p	-0.60286	7.69E-06	down
PAS_chr1-1_0238	Protein whose overexpression affects chromosome stability, potential Cdc28p substrate	-0.75119	1.94E-07	down
PAS_chr1-1_0242	DNA Topoisomerase III, conserved protein that functions in a complex with Sgs1p and Rmi1p	-0.6094	0.018291	down
PAS_chr1-1_0246	Hypothetical protein	-0.93828	0.00075	down
PAS_chr1-1_0250	Essential component of the conserved oligomeric Golgi complex (Cog1p through Cog8p)	-0.66165	0.007385	down
PAS_chr1-1_0251	Hypothetical protein	-0.94171	0.002985	down
PAS_chr1-1_0260	Hypothetical protein	-0.8069	8.05E-07	down
PAS_chr1-1_0288	Subunit of the RNA polymerase II mediator complex	-0.60786	0.027485	down
PAS_chr1-1_0291	Protein required for ribosomal large subunit maturation, functionally redundant with Ssf1p	-0.80622	0.000345	down
PAS_chr1-1_0296	Mitochondrial ribosomal protein of the small subunit	-0.65841	4.21E-05	down
PAS_chr1-1_0297	Mitochondrial protein kinase	-0.84151	3.59E-08	down
PAS_chr1-1_0326	Hypothetical protein	-0.6156	1.47E-05	down
PAS_chr1-1_0331	90S preribosome/SSU processome component KRR1	-0.69294	7.48E-06	down
PAS_chr1-1_0332	Pheromone-regulated multispanning membrane protein involved in membrane fusion during mating	-1.10614	6.45E-09	down
PAS_chr1-1_0367	Protein that interacts with Cdc48p and Npl4p, involved in recognition of polyubiquitinated proteins	-0.80649	8.21E-13	down
PAS_chr1-1_0374	Conserved NAD+ dependent histone deacetylase of the Sirtuin family	-0.61358	3.58E-06	down
PAS_chr1-1_0377	RNA exonuclease	-0.62941	1.37E-06	down
PAS_chr1-1_0381	bZIP transcription factor (ATF/CREB1 homolog) that regulates the unfolded protein response	-0.785	3.71E-13	down
PAS_chr1-1_0393	Chitin synthase I	-0.77365	3.66E-09	down
PAS_chr1-1_0399	Hypothetical protein	-0.82653	4.32E-14	down
PAS_chr1-1_0404	Putative integral membrane protein of unknown function	-0.70049	4.47E-07	down
PAS_chr1-1_0406	Hypothetical protein	-0.84315	0.000115	down
PAS_chr1-1_0407	Putative protein of unknown function	-0.6464	1.05E-07	down
PAS_chr1-1_0427	Putative protein of unknown function with some similarity to GPM1/YKL152C, a phosphoglycerate mutase	-0.79284	3.25E-07	down
PAS_chr1-1_0444	Cytoplasmic protein required for sporulation	-0.61163	0.010675	down
PAS_chr1-1_0446	Protein involved in an early, nucleolar step of 60S ribosomal subunit biogenesis	-0.60425	5.33E-05	down
PAS_chr1-1_0450	Subunit of a complex with Ctf8p and Ctf18p that shares some components with Replication Factor C	-0.98807	1.17E-17	down
PAS_chr1-1_0457	Phosphatidylinositol-4-phosphate 5-kinase	-0.84921	1.01E-10	down
PAS_chr1-1_0468	hypothetical protein	-1.29821	1.90E-05	down
PAS_chr1-1_0473	hypothetical protein	-0.70583	4.75E-07	down
PAS_chr1-1_0474	hypothetical protein	-0.89352	1.49E-07	down
PAS_chr1-1_0477	hypothetical protein	-0.59928	0.059758	down
PAS_chr1-1_0481	hypothetical protein	-0.86362	1.90E-06	down
PAS_chr1-1_0493	hypothetical protein	-0.64636	0.000805	down
PAS_chr1-3_0014	Flavin-containing monooxygenase, localized to the cytoplasmic face of the ER membrane	-0.75946	1.72E-08	down
PAS_chr1-3_0020	Hypothetical protein	-0.58768	2.73E-07	down
PAS_chr1-3_0055	Class II abasic (AP) endonuclease involved in repair of	-0.89651	0.000651	down

	DNA damage			
PAS_chr1-3_0074	Cytoplasmic ATP-dependent RNA helicase of the DEAD-box family	-0.99301	1.75E-14	down
PAS_chr1-3_0079	Nuclear protein that inhibits replication of Brome mosaic virus in S. cerevisiae	-0.69649	3.16E-08	down
PAS_chr1-3_0080	Hydrophilic protein that acts in conjunction with SNARE proteins	-0.93784	5.63E-06	down
PAS_chr1-3_0088	Hypothetical protein	-0.85835	0.000207	down
PAS_chr1-3_0093	Protein with similarity to bovine phospholipase A1	-0.90197	5.50E-06	down
PAS_chr1-3_0118	Mitochondrial ribosomal protein of the large subunit	-1.0639	7.05E-14	down
PAS_chr1-3_0130	Pyrimidine nucleotidase	-0.64756	5.07E-08	down
PAS_chr1-3_0174	One of several homologs of bacterial chaperone DnaJ, located in the ER lumen	-0.77772	5.85E-06	down
PAS_chr1-3_0179	Hypothetical protein	-0.71775	2.14E-07	down
PAS_chr1-3_0182	Essential splicing factor	-0.75283	0.001452	down
PAS_chr1-3_0184	Protein involved in proteolytic activation of Rim101p in response to alkaline pH	-0.67452	1.72E-06	down
PAS_chr1-3_0192	Alpha subunit of both the farnesyltransferase and type I geranylgeranyltransferase	-0.77702	0.000313	down
PAS_chr1-3_0195	Essential, non-ATPase regulatory subunit of the 26S proteasome	-1.07461	2.99E-35	down
PAS_chr1-3_0204	Putative protein of unknown function with strong similarity to alanyl-tRNA synthases from Eubacteria	-0.76402	1.85E-09	down
PAS_chr1-3_0219	Cis-golgi GTPase-activating protein (GAP)	-0.62391	0.005421	down
PAS_chr1-3_0242	Component of the evolutionarily conserved kinetochore-associated Ndc80 complex	-0.64635	0.033413	down
PAS_chr1-3_0260	Hypothetical protein	-0.81044	3.39E-07	down
PAS_chr1-3_0269	Proline oxidase, nuclear-encoded mitochondrial protein	-0.74154	0.000535	down
PAS_chr1-3_0272	Putative ATP-dependent RNA helicase, nucleolar protein	-0.65077	0.00011	down
PAS_chr1-3_0278	Hypothetical protein	-0.7605	0.004953	down
PAS_chr1-3_0279	Hypothetical protein	-1.28563	5.51E-09	down
PAS_chr1-3_0288	Hypothetical protein	-0.65485	0.040516	down
PAS_chr1-3_0289	Hypothetical protein	-0.71605	1.80E-08	down
PAS_chr1-3_0291	hypothetical protein	-0.98288	0.002612	down
PAS_chr1-3_0298	hypothetical protein	-0.69197	0.013939	down
PAS_chr1-3_0311	hypothetical protein	-1.03855	0.000359	down
PAS_chr1-3_0312	hypothetical protein	-0.61793	0.014199	down
PAS_chr1-4_0010	Beta-adaptin, large subunit of the clathrin-associated protein (AP-1) complex	-0.73278	8.05E-10	down
PAS_chr1-4_0021	CBK1 kinase activator protein MOB2	-0.68623	0.000142	down
PAS_chr1-4_0023	One of four subunits of the endosomal sorting complex required for transport III (ESCRT-III)	-0.75591	5.03E-10	down
PAS_chr1-4_0024	cis-Golgi t-SNARE syntaxin required for vesicular transport between the ER and the Golgi complex	-0.63298	0.001278	down
PAS_chr1-4_0029	tRNA-specific 2-thiouridylase, responsible for 2-thiolation of the wobble base of mitochondrial tRNA	-0.6885	0.000538	down
PAS_chr1-4_0037	Alpha-1,2-mannosyltransferase	-0.72078	6.74E-09	down
PAS_chr1-4_0038	Transcription elongation factor S-II	-0.69424	0.000179	down
PAS_chr1-4_0040	High affinity iron permease involved in the transport of iron across the plasma membrane	-0.92446	2.31E-17	down
PAS_chr1-4_0052	Protein of unknown function that may interact with ribosomes	-0.58754	1.27E-05	down
PAS_chr1-4_0062	hypothetical protein	-0.85954	5.36E-15	down
PAS_chr1-4_0068	Putative protein of unknown function	-0.71974	1.29E-06	down
PAS_chr1-4_0072	Co-chaperone that binds to Hsp82p and activates its ATPase activity	-1.27503	1.59E-09	down
PAS_chr1-4_0073	Hypothetical protein	-0.69969	2.38E-08	down
PAS_chr1-4_0090	Polyamine transport protein specific for spermine	-0.91762	1.80E-14	down
PAS_chr1-4_0094	GTP binding protein	-0.66909	0.000148	down
PAS_chr1-4_0106	Arginine methyltransferase	-0.73383	3.72E-06	down
PAS_chr1-4_0109	Protein involved in iron metabolism in mitochondria	-0.85009	1.11E-15	down
PAS_chr1-4_0119	Essential protein involved in the TOR signaling	-0.8184	3.58E-05	down

	pathway			
PAS_chr1-4_0123	Putative protein of unknown function	-0.82646	9.19E-13	down
PAS_chr1-4_0130	Heat shock protein Hsp90	-1.16754	2.88E-28	down
PAS_chr1-4_0134	Core component of the signal recognition particle (SRP) ribonucleoprotein (RNP) complex	-0.67182	4.35E-10	down
PAS_chr1-4_0148	Mitochondrial ribosomal protein of the large subunit	-0.98802	1.25E-10	down
PAS_chr1-4_0158	Clathrin light chain	-0.6184	9.38E-13	down
PAS_chr1-4_0170	Hypothetical protein	-0.66702	0.001016	down
PAS_chr1-4_0188	Nitric oxide oxidoreductase, flavohemoglobin involved in nitric oxide detoxification	-1.40695	1.55E-31	down
PAS_chr1-4_0191	ATP-dependent RNA helicase, component of the mitochondrial degradosome along with the RNase Dss1p	-0.77037	5.13E-05	down
PAS_chr1-4_0196	Phosphatidylinositol 3,5-bisphosphate-binding protein	-0.59775	3.80E-05	down
PAS_chr1-4_0198	Pantothenate synthase, also known as pantoate-beta-alanine ligase	-0.88786	4.98E-18	down
PAS_chr1-4_0210	Core component of the signal recognition particle (SRP) ribonucleoprotein (RNP) complex	-0.70507	0.00035	down
PAS_chr1-4_0224	Hypothetical protein	-0.83675	9.73E-08	down
PAS_chr1-4_0227	hypothetical protein	-0.89864	1.21E-05	down
PAS_chr1-4_0263	Protein required for maturation of the 25S and 5.8S ribosomal RNAs	-0.84359	2.19E-11	down
PAS_chr1-4_0318	Transcription initiation factor TFIID subunit 3	-0.68179	0.02861	down
PAS_chr1-4_0327	Hypothetical protein	-1.08247	0.006567	down
PAS_chr1-4_0335	Conserved nuclear RNA-binding protein	-1.14007	3.90E-19	down
PAS_chr1-4_0342	Essential protein with similarity to phosducins, which are G-protein regulators	-0.7264	2.76E-05	down
PAS_chr1-4_0359	RNA polymerase II subunit B12.6	-1.51163	0.000442	down
PAS_chr1-4_0361	Hypothetical protein	-0.59629	2.76E-09	down
PAS_chr1-4_0367	C-5 sterol desaturase, catalyzes the introduction of a C-5(6) double bond into episterol	-1.04545	2.17E-21	down
PAS_chr1-4_0385	Ser/Thr protein kinase involved in salt tolerance	-0.72292	4.32E-06	down
PAS_chr1-4_0403	Essential evolutionarily-conserved nucleolar protein component of the box C/D snoRNP complexes	-0.63534	9.10E-11	down
PAS_chr1-4_0405	Subunit of the heterohexameric Gim/prefoldin protein complex	-0.64648	0.034345	down
PAS_chr1-4_0409	Putative positive regulator of mannosylphosphate transferase (Mnn6p)	-0.59896	0.000362	down
PAS_chr1-4_0446	Hypothetical protein	-0.59628	6.77E-05	down
PAS_chr1-4_0450	N-succinyl-5-aminoimidazole-4-carboxamide ribotide (SAICAR) synthetase	-0.78036	4.64E-13	down
PAS_chr1-4_0462	Membrane glycoprotein v-SNARE	-0.60655	0.000452	down
PAS_chr1-4_0463	Cytosolic seryl-tRNA synthetase, class II aminoacyl-tRNA synthetase	-0.59484	2.25E-12	down
PAS_chr1-4_0492	Transcriptional modulator	-0.68726	1.71E-07	down
PAS_chr1-4_0493	Nucleolar protein, required for pre-25S rRNA processing	-0.66692	0.002318	down
PAS_chr1-4_0526	Regulatory protein MIG1	-0.60338	1.21E-06	down
PAS_chr1-4_0529	Subunit of the heterodimeric FACT complex (Spt16p-Pob3p)	-0.79597	6.51E-19	down
PAS_chr1-4_0539	Essential nucleolar protein involved in pre-18S rRNA processing	-0.665	0.000906	down
PAS_chr1-4_0596	Subunit of the RNA polymerase II-associated Paf1 complex	-0.80951	5.60E-08	down
PAS_chr1-4_0606	Membrane-associated protein	-0.68424	0.00439	down
PAS_chr1-4_0614	Core Sm protein Sm D3	-0.6633	0.020484	down
PAS_chr1-4_0633	5' to 3' exonuclease, 5' flap endonuclease	-0.94604	2.71E-10	down
PAS_chr1-4_0635	Hypothetical protein	-0.6028	0.004825	down
PAS_chr1-4_0639	transcription factor	-0.59459	2.21E-05	down
PAS_chr1-4_0640	U3 small nucleolar RNA-associated protein	-1.0078	6.62E-14	down
PAS_chr1-4_0649	Mitochondrial respiratory chain complexes assembly protein RCA1	-0.81052	3.43E-13	down
PAS_chr1-4_0650	Chromatin structure-remodeling complex protein RSC7	-0.63586	7.52E-07	down

PAS_chr1-4_0653	hypothetical protein	-0.963	1.85E-13	down
PAS_chr1-4_0656	hypothetical protein	-0.74992	1.27E-07	down
PAS_chr1-4_0662	hypothetical protein	-0.69157	0.000232	down
PAS_chr1-4_0663	hypothetical protein	-0.65639	1.87E-09	down
PAS_chr1-4_0678	hypothetical protein	-0.61143	0.075371	down
PAS_chr1-4_0693	hypothetical protein	-0.88937	2.86E-07	down
PAS_chr2-1_0011	ATPase of the ATP-binding cassette (ABC) family involved in 40S and 60S ribosome biogenesis, has sim	-0.87329	1.44E-07	down
PAS_chr2-1_0027	Putative protein of unknown function	-0.88713	4.03E-07	down
PAS_chr2-1_0036	Hypothetical protein	-0.6296	0.001953	down
PAS_chr2-1_0039	Essential 121kDa subunit of the exocyst complex (Sec3p, Sec5p, Sec6p, Sec8p, Sec10p, Sec15p, Exo70p	-0.68964	0.00021	down
PAS_chr2-1_0040	Protein with similarity to ATP-binding cassette (ABC) transporter family members	-0.74493	1.31E-07	down
PAS_chr2-1_0042	Hsp70 (Ssa1p) nucleotide exchange factor, cytosolic homolog of Sil1p, which is the nucleotide exchan	-0.63755	1.22E-06	down
PAS_chr2-1_0043	Subunit of TFIIH complex, involved in transcription initiation, similar to 34 kDa subunit of human T	-0.65655	2.11E-08	down
PAS_chr2-1_0056	Palmitoyltransferase that acts on the SNAREs Snc1p, Syn8p, Tlg1p and likely on all SNAREs	-0.64388	0.066635	down
PAS_chr2-1_0061	Checkpoint protein, involved in the activation of the DNA damage and meiotic pachytene checkpoints	-0.7035	0.000908	down
PAS_chr2-1_0062	JmjC domain-containing histone demethylase which can specifically demethylate H3K36 tri-and dimethy	-0.65169	6.83E-05	down
PAS_chr2-1_0067	Pseudouridine synthase, catalyzes only the formation of pseudouridine-55 (Psi55), a highly conserved	-0.80719	0.006147	down
PAS_chr2-1_0074	GTPase-activating protein for Sec4p and several other Rab GTPases, regulates exocytosis via its acti	-1.03701	1.03E-12	down
PAS_chr2-1_0081	Hypothetical protein	-0.70616	0.000179	down
PAS_chr2-1_0101	Exopolyphosphatase, hydrolyzes inorganic polyphosphate (poly P) into Pi residues	-1.15324	1.12E-13	down
PAS_chr2-1_0139	Mitochondrial ribosomal protein of the small subunit	-0.61935	0.000166	down
PAS_chr2-1_0153	Protein that stimulates strand exchange by facilitating Rad51p binding to single-stranded DNA	-1.01756	2.12E-22	down
PAS_chr2-1_0185	Protein subunit of mitochondrial RNase P, has roles in nuclear transcription, cytoplasmic and mitoch	-0.89225	5.79E-16	down
PAS_chr2-1_0187	Hypothetical protein	-0.83787	4.10E-07	down
PAS_chr2-1_0200	F-box receptor protein, subunit of the Skp1-Cdc53-F-box receptor (SCF) E3 ubiquitin ligase complex	-0.9717	2.52E-10	down
PAS_chr2-1_0206	Subunit of COMPASS (Set1C), a complex which methylates histone H3 on lysine 4 and is required in tel	-1.01054	2.42E-09	down
PAS_chr2-1_0207	Mitochondrial GTPase related to dynamin, present in a complex containing Ugo1p and Fzo1p	-0.87648	2.29E-16	down
PAS_chr2-1_0232	Cleavage and polyadenylation factor I (CF I) component involved in cleavage and polyadenylation of m	-0.60547	3.29E-05	down
PAS_chr2-1_0244	Mitochondrial translation initiation factor 2	-0.72572	0.041096	down
PAS_chr2-1_0266	Hypothetical protein	-0.97104	0.059705	down
PAS_chr2-1_0274	Protein involved in postreplication repair	-0.59127	0.008433	down
PAS_chr2-1_0276	Hypothetical protein	-0.74109	1.03E-07	down
PAS_chr2-1_0277	TFIIA large subunit	-0.88935	7.23E-10	down
PAS_chr2-1_0278	Hypothetical protein	-1.00996	1.56E-06	down
PAS_chr2-1_0283	Putative tRNA acetyltransferase	-0.60257	0.004867	down
PAS_chr2-1_0288	Hypothetical protein	-0.64408	0.001454	down
PAS_chr2-1_0324	Oligomeric mitochondrial matrix chaperone	-0.83392	5.61E-17	down
PAS_chr2-1_0339	Putative protein of unknown function	-0.74527	8.43E-15	down
PAS_chr2-1_0340	Hypothetical protein	-0.60464	0.022664	down
PAS_chr2-1_0344	Protein required for assembly of cytochrome c oxidase	-0.91293	0.000342	down
PAS_chr2-1_0347	Protein kinase	-0.80747	3.11E-08	down
PAS_chr2-1_0348	Hypothetical protein	-0.65008	0.01637	down
PAS_chr2-1_0353	Hypothetical protein	-0.89858	0.01303	down
PAS_chr2-1_0360	Putative protein with sequence similarity to hydroxyacid dehydrogenases	-0.85009	0.00501	down
PAS_chr2-1_0369	Ubiquitin-protein ligase, member of the cullin family	-0.62556	1.05E-05	down

	with similarity to Cdc53p and human CUL3			
PAS_chr2-1_0379	Signal recognition particle (SRP) receptor-alpha subunit	-0.75071	3.30E-11	down
PAS_chr2-1_0387	Subunit of RNase MRP	-0.97197	0.00016	down
PAS_chr2-1_0396	Mitochondrial methionyl-tRNA synthetase (MetRS)	-0.75269	1.19E-05	down
PAS_chr2-1_0398	DNA repair protein Nse1	-0.60516	0.008706	down
PAS_chr2-1_0405	Hypothetical protein	-0.70185	1.84E-05	down
PAS_chr2-1_0414	Cytoplasmic RNA-binding protein, contains an RNA recognition motif (RRM)	-0.622	3.29E-05	down
PAS_chr2-1_0421	ER protein with chaperone and co-chaperone activity, involved in retention of resident ER proteins	-0.6884	1.27E-06	down
PAS_chr2-1_0430	Essential subunit of the COMPASS (Set1C) complex	-0.85659	0.000587	down
PAS_chr2-1_0436	Putative RNA binding protein	-0.64548	1.83E-06	down
PAS_chr2-1_0443	Hypothetical protein	-1.13841	0.000145	down
PAS_chr2-1_0444	Hypothetical protein	-0.75265	1.41E-09	down
PAS_chr2-1_0455	Protein required for assembly of U2 snRNP into the spliceosome, forms a complex with Hsh49p and Hsh1	-0.64778	0.001669	down
PAS_chr2-1_0458	Peroxin 20	-0.99574	1.68E-09	down
PAS_chr2-1_0499	RNA binding protein required for maturation of tRNA and snRNA precursors	-0.75954	0.006084	down
PAS_chr2-1_0509	Putative protein of unknown function	-0.70283	2.43E-10	down
PAS_chr2-1_0528	Hypothetical protein	-0.85721	0.002699	down
PAS_chr2-1_0544	Hypothetical protein	-1.01161	0.006492	down
PAS_chr2-1_0572	AAA-type ATPase that is regulated by Vta1p	-0.72193	6.77E-07	down
PAS_chr2-1_0574	Component of CORVET tethering complex	-0.60151	0.004458	down
PAS_chr2-1_0585	Cis-golgi localized monothiol glutaredoxin that binds an iron-sulfur cluster	-0.89359	1.89E-13	down
PAS_chr2-1_0590	Cytoplasmic protein of unknown function predicted to encode a DNA-3-methyladenine glycosidase II	-0.6013	0.001917	down
PAS_chr2-1_0595	Calcineurin B	-0.64802	0.046803	down
PAS_chr2-1_0597	Hypothetical protein	-0.62651	8.71E-08	down
PAS_chr2-1_0601	Conserved nuclear regulatory subunit of Glc7p type 1 protein serine-threonine phosphatase (PP1)	-0.86361	1.86E-14	down
PAS_chr2-1_0605	Putative Nedd8 ligase	-1.08216	5.87E-10	down
PAS_chr2-1_0611	DNA Polymerase phi	-0.65879	2.25E-11	down
PAS_chr2-1_0613	Mitochondrial inner membrane half-type ATP-binding cassette (ABC) transporter	-0.79283	2.43E-06	down
PAS_chr2-1_0617	Nuclear pore protein involved in nuclear export of pre-tRNA	-0.62905	2.62E-05	down
PAS_chr2-1_0627	Non-SMC subunit of the condensin complex (Smc2p-Smc4p-Ycs4p-Brn1p-Ycg1p)	-0.76229	2.82E-05	down
PAS_chr2-1_0629	Protein involved in transcription-coupled repair nucleotide excision repair of UV-induced DNA lesion	-0.60611	9.09E-06	down
PAS_chr2-1_0630	Microtubule effector required for tubulin heterodimer formation	-0.72165	0.005155	down
PAS_chr2-1_0642	Hypothetical protein	-0.73953	1.58E-10	down
PAS_chr2-1_0653	Mitochondrial cruciform cutting endonuclease	-1.2171	2.42E-06	down
PAS_chr2-1_0665	Hypothetical protein	-0.59475	0.000233	down
PAS_chr2-1_0672	Scaffold protein responsible for pre-autophagosomal structure organization	-0.59914	0.015449	down
PAS_chr2-1_0674	Constituent of Paf1 complex with RNA polymerase II, Paf1p, Hpr1p, Ctr9, Leo1, Rtf1 and Ccr4p	-0.80588	3.26E-09	down
PAS_chr2-1_0683	Mitochondrial ribosomal protein of the small subunit	-0.92341	6.62E-08	down
PAS_chr2-1_0688	Essential protein involved in transcription regulation	-0.60851	1.15E-10	down
PAS_chr2-1_0697	Catalytic subunit of DNA polymerase zeta, which is involved in DNA repair and translesion synthesis	-0.74152	3.85E-05	down
PAS_chr2-1_0702	Protein that recognizes and binds damaged DNA during nucleotide excision repair	-0.7051	2.15E-07	down
PAS_chr2-1_0704	Protein of unknown function	-0.8843	0.002543	down
PAS_chr2-1_0706	Putative protein of unknown function	-0.79948	1.35E-15	down
PAS_chr2-1_0708	Mitochondrial inner membrane protein	-0.69425	0.000187	down
PAS_chr2-1_0711	Hypothetical protein	-0.66366	0.033403	down
PAS_chr2-1_0720	Component of both the SWI/SNF and RSC chromatin	-0.83941	3.88E-14	down

	remodeling complexes			
PAS_chr2-1_0724	Hypothetical protein	-1.14953	0.000493	down
PAS_chr2-1_0737	Hypothetical protein	-0.81558	9.83E-20	down
PAS_chr2-1_0741	Translation initiation factor eIF1A	-0.69115	2.10E-05	down
PAS_chr2-1_0748	Hypothetical protein	-0.64423	3.15E-13	down
PAS_chr2-1_0753	Alpha-1,2-mannosidase involved in ER quality control	-0.89811	1.03E-14	down
PAS_chr2-1_0759	Mannosyltransferase, involved in asparagine-linked glycosylation in the endoplasmic reticulum (ER)	-0.73299	4.55E-10	down
PAS_chr2-1_0774	Subunit of TRAPP (transport protein particle)	-1.1234	1.11E-22	down
PAS_chr2-1_0781	Nucleolar protein, component of the small subunit (SSU) processome	-0.7253	4.51E-07	down
PAS_chr2-1_0789	Subunit of the membrane-associated retromer complex essential for endosome-to-Golgi retrograde prote	-0.60572	0.000182	down
PAS_chr2-1_0790	Hypothetical protein	-0.62102	0.015498	down
PAS_chr2-1_0796	Protein localized to COPII-coated vesicles, forms a complex with Erv41p	-0.80811	1.10E-12	down
PAS_chr2-1_0818	hypothetical protein	-0.84784	0.013528	down
PAS_chr2-1_0821	hypothetical protein	-0.74049	5.11E-08	down
PAS_chr2-1_0825	hypothetical protein	-0.6172	6.44E-10	down
PAS_chr2-1_0832	hypothetical protein	-0.64036	0.000128	down
PAS_chr2-1_0838	hypothetical protein	-0.80677	0.000991	down
PAS_chr2-1_0845	hypothetical protein	-0.77307	6.60E-08	down
PAS_chr2-1_0854	hypothetical protein	-0.71864	3.78E-06	down
PAS_chr2-1_0870	hypothetical protein	-0.65449	0.002266	down
PAS_chr2-1_0883	hypothetical protein	-0.78274	0.006118	down
PAS_chr2-2_0012	Hypothetical protein	-0.65608	0.030879	down
PAS_chr2-2_0015	One of several homologs of bacterial chaperone DnaJ, located in the ER lumen	-0.72277	0.081553	down
PAS_chr2-2_0018	Protein of unknown function	-0.78433	1.50E-06	down
PAS_chr2-2_0031	Protein of unknown function	-1.04715	0.009988	down
PAS_chr2-2_0051	Putative protein of unknown function	-0.69769	8.91E-07	down
PAS_chr2-2_0068	Nuclear actin-related protein involved in chromatin remodeling	-0.73216	4.54E-16	down
PAS_chr2-2_0072	Protein of unknown function that may interact with ribosomes	-0.61367	1.71E-12	down
PAS_chr2-2_0085	Cytosolic J-domain-containing protein	-0.90306	7.23E-10	down
PAS_chr2-2_0092	Chaperone that specifically facilitates the assembly of cytochrome c oxidase	-0.62399	0.004947	down
PAS_chr2-2_0096	Integral inner mitochondrial membrane protein	-0.66149	1.94E-13	down
PAS_chr2-2_0106	Possible U3 snoRNP protein	-0.84137	0.007853	down
PAS_chr2-2_0122	Mitochondrial matrix protein	-0.68128	4.29E-08	down
PAS_chr2-2_0126	Presequence translocase-associated motor subunit	-0.65531	6.33E-08	down
PAS_chr2-2_0130	Essential protein involved in maturation of 18S rRNA	-0.86805	0.002887	down
PAS_chr2-2_0151	Type II HSP40 co-chaperone that interacts with the HSP70 protein Ssa1p	-0.7468	5.01E-10	down
PAS_chr2-2_0152	Hypothetical protein	-0.64594	2.65E-07	down
PAS_chr2-2_0166	JmjC domain family histone demethylase	-0.68925	1.07E-09	down
PAS_chr2-2_0172	Protein that interacts with mitotic cyclin Clb2p	-0.6558	6.97E-13	down
PAS_chr2-2_0189	Putative protein of unknown function with similarity to proteins containing WD-40 domains	-0.86533	0.000387	down
PAS_chr2-2_0190	Essential subunit of RNA polymerase III transcription factor (TFIIIB), which is involved in transcri	-0.72968	0.00013	down
PAS_chr2-2_0208	Hypothetical protein	-0.66241	1.08E-11	down
PAS_chr2-2_0209	Protein with similarity to tRNA synthetases	-0.72904	0.000579	down
PAS_chr2-2_0215	3'--&gt5' exonuclease and endonuclease with a possible role in apoptosis	-1.22348	1.28E-13	down
PAS_chr2-2_0216	Type 2C protein phosphatase	-0.73912	2.79E-17	down
PAS_chr2-2_0227	Subunit d of the five-subunit V0 integral membrane domain of vacuolar H+-ATPase (V-ATPase)	-0.82332	1.58E-13	down
PAS_chr2-2_0246	Subunit of the HIR complex, a nucleosome assembly	-0.70151	4.04E-07	down

	complex involved in histone gene transcription			
PAS_chr2-2_0250	Subunit of the tRNA splicing endonuclease, which is composed of Sen2p, Sen15p, Sen34p, and Sen54p	-0.59074	0.046128	down
PAS_chr2-2_0261	Hypothetical protein	-0.69264	1.10E-06	down
PAS_chr2-2_0284	Transcriptional regulator involved in glucose repression of Gal4p-regulated genes	-0.72456	9.02E-08	down
PAS_chr2-2_0285	Hypothetical protein	-0.66788	5.88E-08	down
PAS_chr2-2_0312	Hypothetical protein	-0.6285	0.020319	down
PAS_chr2-2_0321	Hypothetical protein	-0.80165	3.18E-06	down
PAS_chr2-2_0324	Hypothetical protein	-0.6757	5.31E-07	down
PAS_chr2-2_0341	Metalloprotease subunit of the 19S regulatory particle of the 26S proteasome lid	-0.79284	7.21E-17	down
PAS_chr2-2_0347	GTP-binding protein of the ras superfamily required for bud site selection	-0.85615	4.71E-10	down
PAS_chr2-2_0369	Hypothetical protein	-1.04631	0.018473	down
PAS_chr2-2_0373	Protein with a role in urmylation and in invasive and pseudohyphal growth	-0.80898	1.80E-05	down
PAS_chr2-2_0376	Hypothetical protein	-0.74802	9.52E-06	down
PAS_chr2-2_0401	Cholinephosphate cytidylyltransferase, also known as CTP:phosphocholine cytidylyltransferase	-0.83892	4.35E-08	down
PAS_chr2-2_0424	Splicing factor, component of the U4/U6-U5 snRNP complex	-0.64195	0.003854	down
PAS_chr2-2_0434	RNA polymerase subunit ABC23, common to RNA polymerases I, II, and III	-0.83422	4.15E-06	down
PAS_chr2-2_0439	Putative protein of unknown function	-1.02585	5.32E-08	down
PAS_chr2-2_0442	Hypothetical protein	-0.75846	1.10E-11	down
PAS_chr2-2_0443	Protein that recognizes and binds damaged DNA in an ATP-dependent manner (with Rad16p)	-0.90125	1.61E-25	down
PAS_chr2-2_0450	hypothetical protein	-0.64239	0.0109	down
PAS_chr2-2_0453	hypothetical protein	-1.3399	1.28E-06	down
PAS_chr2-2_0486	hypothetical protein	-0.70997	0.003535	down
PAS_chr3_0012	Hypothetical protein	-0.58899	0.037987	down
PAS_chr3_0043	peroxisomal integral membrane protein	-0.80002	8.26E-19	down
PAS_chr3_0047	Nuclear actin-related protein involved in chromatin remodeling, component of chromatin-remodeling en	-0.80026	1.12E-05	down
PAS_chr3_0048	Essential t-SNARE that forms a complex with Tlg2p and Vti1p and mediates fusion of endosome-derived	-0.73921	2.90E-07	down
PAS_chr3_0057	Hypothetical protein	-0.59116	0.075767	down
PAS_chr3_0074	Protein that acts together with Uba3p to activate Rub1p before its conjugation to proteins (neddylat	-0.58745	1.29E-06	down
PAS_chr3_0112	Essential serine kinase involved in cell cycle progression and processing of the 20S pre-rRNA into m	-0.84742	0.05323	down
PAS_chr3_0114	Hypothetical protein	-0.78224	1.10E-05	down
PAS_chr3_0118	One of six subunits of RNA polymerase III transcription initiation factor complex (TFIIIC)	-0.58976	0.000553	down
PAS_chr3_0200	Sumoylated protein of unknown function	-0.64152	1.59E-05	down
PAS_chr3_0210	Subunit of the vacuole fusion and protein sorting HOPS complex and the CORVET tethering complex	-0.91932	5.06E-13	down
PAS_chr3_0224	DNA-dependent ATPase	-0.86906	8.05E-17	down
PAS_chr3_0235	Zinc-finger protein of unknown function	-0.68591	3.23E-06	down
PAS_chr3_0240	Essential protein required for biogenesis of 40S (small) ribosomal subunit	-0.84019	1.65E-09	down
PAS_chr3_0242	Topoisomerase I	-0.60003	4.68E-05	down
PAS_chr3_0244	RNA polymerase II subunit B12.5	-0.84438	0.00823	down
PAS_chr3_0262	Thymidylate and uridylate kinase	-0.60179	0.000179	down
PAS_chr3_0263	Mitochondrial outer membrane protein	-0.62698	2.88E-06	down
PAS_chr3_0269	Protein required for sorting proteins to the vacuole	-0.88172	5.68E-06	down
PAS_chr3_0276	Hypothetical protein	-0.76911	0.000679	down
PAS_chr3_0280	Protein of unknown function	-0.68821	3.86E-06	down
PAS_chr3_0312	Component of the ESCRT-II complex	-0.68983	0.000438	down
PAS_chr3_0325	Essential splicesome assembly factor	-0.96312	1.80E-10	down

PAS_chr3_0359	Ubiquitin-conjugating enzyme (E2)	-0.88591	9.43E-09	down
PAS_chr3_0362	Hypothetical protein	-0.99898	1.46E-06	down
PAS_chr3_0374	Hypothetical protein	-0.80079	1.41E-19	down
PAS_chr3_0390	Subunit of RAVE (Rav1p, Rav2p, Skp1p)	-1.29486	4.41E-16	down
PAS_chr3_0399	Minor sphingoid long-chain base kinase	-0.71197	7.17E-09	down
PAS_chr3_0400	Hypothetical protein	-0.8329	3.90E-06	down
PAS_chr3_0426	Protein involved in homologous recombination in mitochondria and in transcription regulation in nucl	-0.72966	9.21E-07	down
PAS_chr3_0431	Subunit of the Nsp1p-Nup57p-Nup49p-Nic96p subcomplex of the nuclear pore complex (NPC)	-0.70177	3.39E-09	down
PAS_chr3_0433	Subunit of the RES complex	-0.73772	0.000765	down
PAS_chr3_0457	Essential, non-ATPase regulatory subunit of the 26S proteasome	-1.14877	2.31E-27	down
PAS_chr3_0472	Hypothetical protein	-0.75698	0.057986	down
PAS_chr3_0477	Hypothetical protein	-0.65019	0.033544	down
PAS_chr3_0480	Putative chaperone, homolog of E. coli DnaJ, closely related to Ydj1p	-0.68798	4.06E-05	down
PAS_chr3_0485	Cytoplasmic protein required for cytoplasm to vacuole targeting of proteins	-0.91462	5.15E-11	down
PAS_chr3_0494	Hypothetical protein	-0.59576	1.33E-10	down
PAS_chr3_0540	Subunit of the cohesin complex	-0.82431	1.80E-16	down
PAS_chr3_0552	UDP-glucose:dolichyl-phosphate glucosyltransferase	-0.8361	4.78E-05	down
PAS_chr3_0603	Protein involved in rRNA processing	-0.9252	1.97E-09	down
PAS_chr3_0608	Protein serine/threonine phosphatase with similarity to human phosphatase PP5	-0.96472	7.18E-11	down
PAS_chr3_0609	Beta (RNA 5'-triphosphatase) subunit of the mRNA capping enzyme	-0.64849	0.000752	down
PAS_chr3_0642	Subunit of the SF3a splicing factor complex, required for spliceosome assembly	-0.8687	0.005089	down
PAS_chr3_0644	RNA helicase in the DEAD-box family, necessary for prespliceosome formation	-0.63672	9.38E-06	down
PAS_chr3_0652	NAP family histone chaperone	-0.83946	0.029003	down
PAS_chr3_0678	Protein that interacts with the karyopherin Srp1p	-0.9902	9.34E-08	down
PAS_chr3_0680	Hypothetical protein	-0.96439	1.32E-05	down
PAS_chr3_0682	Cytoplasmic protein required for replication of Brome mosaic virus in S. cerevisiae	-0.88696	2.92E-09	down
PAS_chr3_0683	Essential nucleolar protein that is a component of the SSU (small subunit) processome	-0.61425	0.011205	down
PAS_chr3_0699	Alpha 7 subunit of the 20S proteasome	-0.6888	8.75E-11	down
PAS_chr3_0723	Hypothetical protein	-0.81322	1.22E-06	down
PAS_chr3_0725	Protein with a role in ubiquinone (Coenzyme Q) biosynthesis	-0.60101	7.49E-10	down
PAS_chr3_0734	Hypothetical protein	-0.65968	4.68E-05	down
PAS_chr3_0737	TFIIE small subunit, involved in RNA polymerase II transcription initiation	-0.64172	0.000219	down
PAS_chr3_0748	Beta 1 subunit of the 20S proteasome, responsible for cleavage after acidic residues in peptides	-0.60968	1.82E-08	down
PAS_chr3_0753	Protein involved in rRNA processing	-1.11357	4.50E-08	down
PAS_chr3_0785	Putative protein of unknown function	-1.06682	0.01062	down
PAS_chr3_0812	Hypothetical protein	-0.71622	3.01E-06	down
PAS_chr3_0871	Hypothetical protein	-0.74277	8.08E-12	down
PAS_chr3_0885	Nucleolar protein, component of the small subunit (SSU) processome containing the U3 snoRNA	-0.9099	2.30E-11	down
PAS_chr3_0903	Nucleolar protein	-0.76427	7.30E-08	down
PAS_chr3_0913	Component of the mitotic spindle that binds to interpolar microtubules	-0.6378	0.001942	down
PAS_chr3_0956	Protein involved in rRNA processing	-0.7211	0.001424	down
PAS_chr3_0989	t-SNARE required for ER membrane fusion and vesicular traffic	-0.78206	1.62E-05	down
PAS_chr3_1006	Essential, conserved, cytoplasmic ATPase	-0.72711	7.46E-08	down
PAS_chr3_1008	Hypothetical protein	-1.27539	1.66E-30	down
PAS_chr3_1012	Ketopantoate hydroxymethyltransferase, required for pantothenic acid biosynthesis	-0.71166	9.87E-05	down

PAS_chr3_1034	Hypothetical protein	-0.63768	6.36E-10	down
PAS_chr3_1035	Component of the Paf1p complex	-0.62408	0.033575	down
PAS_chr3_1042	Catalytic component of the exosome, involved in RNA processing and degradation	-0.59494	1.02E-07	down
PAS_chr3_1052	Putative protein of unknown function	-1.14156	7.15E-16	down
PAS_chr3_1060	Hypothetical protein	-0.59155	0.004756	down
PAS_chr3_1064	Protein with putative serine active lipase domain	-0.75944	1.10E-05	down
PAS_chr3_1068	Protein of unknown function that associates with ribosomes	-0.62625	1.71E-06	down
PAS_chr3_1069	Essential, non-ATPase regulatory subunit of the 26S proteasome lid	-1.0086	1.77E-24	down
PAS_chr3_1073	Peroxisomal membrane protein (PMP)	-0.60824	3.12E-06	down
PAS_chr3_1078	Subunit of the anaphase-promoting complex/cyclosome (APC/C)	-0.95488	2.37E-07	down
PAS_chr3_1091	Protein kinase implicated in activation of the plasma membrane H(+)-ATPase Pma1p	-0.61055	6.66E-08	down
PAS_chr3_1100	Subunit of a tRNA methyltransferase complex composed of Trm8p and Trm82p	-0.60224	0.013458	down
PAS_chr3_1103	Component of the pre-60S pre-ribosomal particle	-0.89504	0.000805	down
PAS_chr3_1110	Mitochondrial tyrosyl-tRNA synthetase	-0.91919	5.34E-14	down
PAS_chr3_1115	Mannose-6-phosphate isomerase, catalyzes the interconversion of fructose-6-P and mannose-6-P	-0.59657	8.40E-11	down
PAS_chr3_1151	hypothetical protein	-0.62097	0.01021	down
PAS_chr3_1152	hypothetical protein	-0.87451	4.51E-07	down
PAS_chr3_1163	hypothetical protein	-0.67866	0.001469	down
PAS_chr3_1165	hypothetical protein	-0.87625	1.01E-06	down
PAS_chr3_1172	hypothetical protein	-0.69563	0.000175	down
PAS_chr3_1177	hypothetical protein	-0.6189	4.21E-13	down
PAS_chr3_1178	hypothetical protein	-0.72791	0.00037	down
PAS_chr3_1186	hypothetical protein	-0.62941	0.000694	down
PAS_chr3_1202	hypothetical protein	-0.96122	4.46E-06	down
PAS_chr3_1209	hypothetical protein	-0.60553	2.54E-12	down
PAS_chr3_1211	hypothetical protein	-1.00359	0.001145	down
PAS_chr3_1213	hypothetical protein	-0.64524	0.000675	down
PAS_chr3_1230	ATG30	-0.69321	2.47E-09	down
PAS_chr3_1251	hypothetical protein	-0.75067	0.003314	down
PAS_chr3_1252	hypothetical protein	-0.72974	2.55E-05	down
PAS_chr3_1253	hypothetical protein	-0.63335	0.011209	down
PAS_chr4_0008	Low-affinity Fe(II) transporter of the plasma membrane	-0.62578	0.005046	down
PAS_chr4_0014	Hypothetical protein	-0.70658	1.81E-13	down
PAS_chr4_0028	Subunit of the GINS complex (Sld5p, Psf1p, Psf2p, Psf3p)	-0.76631	0.003831	down
PAS_chr4_0068	Hypothetical protein	-0.97744	0.003161	down
PAS_chr4_0069	Nucleosome assembly factor, involved in chromatin assembly and disassembly	-0.5942	0.000432	down
PAS_chr4_0073	Component of the conserved oligomeric Golgi complex (Cog1p through Cog8p)	-0.68588	0.000245	down
PAS_chr4_0078	Essential protein with dual roles in spliceosome assembly and exocytosis	-0.68438	9.43E-05	down
PAS_chr4_0125	Hypothetical protein	-1.32966	2.50E-08	down
PAS_chr4_0129	Hypothetical protein	-0.89478	0.000315	down
PAS_chr4_0136	Subunit of the histone deacetylase Rpd3L complex	-0.90139	6.94E-07	down
PAS_chr4_0150	Aspartate kinase (L-aspartate 4-P-transferase)	-0.81637	3.97E-13	down
PAS_chr4_0165	GTPase, Ras-like GTP binding protein involved in the secretory pathway	-0.68127	4.45E-06	down
PAS_chr4_0172	E3 ubiquitin ligase for Rad6p	-0.6048	0.001929	down
PAS_chr4_0217	Protein of unknown function that associates with ribosomes	-1.2494	2.62E-26	down
PAS_chr4_0225	Gamma subunit of coatomer, a heptameric protein complex that together with Arf1p forms the COPI coat	-0.64022	1.53E-12	down

PAS_chr4_0229	Catalytic subunit of TRAMP (Trf4/Pap2p-Mtr4p-Air1p/2p)	-0.59191	0.01949	down
PAS_chr4_0230	Hypothetical protein	-1.33901	0.009009	down
PAS_chr4_0231	Putative nucleolar DEAD box RNA helicase	-0.77796	2.32E-09	down
PAS_chr4_0242	Nuclear type II J heat shock protein of the E. coli dnaJ family	-0.83848	1.25E-12	down
PAS_chr4_0244	Hypothetical protein	-0.75897	0.002355	down
PAS_chr4_0257	Putative protein of unknown function	-0.59403	8.32E-06	down
PAS_chr4_0278	Protein involved in pre-rRNA processing, 18S rRNA synthesis, and snoRNA synthesis	-0.77113	6.08E-07	down
PAS_chr4_0293	Component of the septin ring of the mother-bud neck that is required for cytokinesis	-0.87916	1.95E-12	down
PAS_chr4_0299	Hypothetical protein	-0.70852	0.004449	down
PAS_chr4_0317	Hypothetical protein	-0.66933	0.002191	down
PAS_chr4_0319	N-acetyltransferase, confers resistance to the sphingolipid biosynthesis inhibitor myriocin (ISP-1)	-0.98849	3.30E-06	down
PAS_chr4_0320	Hypothetical protein	-0.60793	1.09E-10	down
PAS_chr4_0387	One of six ATPases of the 19S regulatory particle of the 26S proteasome	-1.02025	8.75E-28	down
PAS_chr4_0388	Mitochondrial ribosomal protein of the large subunit	-0.8443	9.43E-05	down
PAS_chr4_0389	Large subunit of the dynactin complex	-0.62057	0.009848	down
PAS_chr4_0426	Hypothetical protein	-0.93303	1.07E-06	down
PAS_chr4_0435	Subunit of the RNA polymerase II mediator complex	-1.37582	0.000124	down
PAS_chr4_0436	RING finger containing subunit of Skp1-Cullin-F-box ubiquitin protein ligases (SCF)	-0.75105	2.06E-05	down
PAS_chr4_0438	Heat shock protein with a zinc finger motif	-0.74694	1.05E-05	down
PAS_chr4_0442	Subunit of the heme-activated, glucose-repressed Hap2p/3p/4p/5p CCAAT-binding complex	-0.97662	2.53E-07	down
PAS_chr4_0450	Hypothetical protein	-0.76478	1.96E-10	down
PAS_chr4_0451	Hypothetical protein	-0.73334	1.41E-12	down
PAS_chr4_0502	Putative protein of unknown function	-0.72646	0.055815	down
PAS_chr4_0518	TFIIF (Transcription Factor II) largest subunit	-0.71921	8.65E-09	down
PAS_chr4_0520	Hypothetical protein	-0.82049	0.000163	down
PAS_chr4_0538	Splicing factor, component of the U4/U6-U5 snRNP complex	-0.71175	0.000914	down
PAS_chr4_0541	Protein that forms a complex with the Sit4p protein phosphatase and is required for its function	-0.77132	3.03E-05	down
PAS_chr4_0547	Hypothetical protein	-0.72057	0.060161	down
PAS_chr4_0548	Component of the septin ring of the mother-bud neck that is required for cytokinesis	-0.64907	6.21E-09	down
PAS_chr4_0549	Hypothetical protein	-0.6965	9.37E-06	down
PAS_chr4_0597	Hypothetical protein	-0.75467	1.61E-09	down
PAS_chr4_0599	Subunit of a possibly tetrameric trichostatin A-sensitive class II histone deacetylase complex	-0.8253	0.0003	down
PAS_chr4_0606	Guanine nucleotide exchange factor (GEF)	-0.64202	3.51E-09	down
PAS_chr4_0615	Putative S-adenosylmethionine-dependent methyltransferase of the seven beta-strand family	-0.61197	0.000565	down
PAS_chr4_0629	GTPase-activating protein (RhoGAP) for Cdc42p and Rho5p	-1.04073	4.08E-10	down
PAS_chr4_0631	Hypothetical protein	-0.62152	0.000585	down
PAS_chr4_0650	Hypothetical protein	-0.83107	1.97E-05	down
PAS_chr4_0653	Protein integral to the mitochondrial membrane	-0.73471	6.80E-07	down
PAS_chr4_0659	Subunit of the RNA polymerase II mediator complex	-0.6805	0.000121	down
PAS_chr4_0661	66S preribosome component MAK16	-0.7002	0.000327	down
PAS_chr4_0670	Hypothetical protein	-0.8569	0.027565	down
PAS_chr4_0700	Phospholipase C	-0.60177	0.020597	down
PAS_chr4_0706	Hypothetical protein	-0.7054	0.000833	down
PAS_chr4_0716	Nucleolar protein involved in rRNA processing and 60S ribosomal subunit biogenesis	-0.60549	7.59E-05	down
PAS_chr4_0717	Essential conserved protein that is part of the 90S preribosome	-0.83431	4.15E-07	down
PAS_chr4_0719	GTPase activating protein (GAP) for Gsp1p, involved in nuclear transport	-0.65943	3.86E-11	down

PAS_chr4_0720	Transcriptional coactivator	-0.86178	1.76E-15	down
PAS_chr4_0722	Hypothetical protein	-0.68327	0.012087	down
PAS_chr4_0729	Putative protein of unknown function	-0.66044	0.027721	down
PAS_chr4_0736	One of six ATPases of the 19S regulatory particle of the 26S proteasome	-0.97298	6.38E-21	down
PAS_chr4_0745	Subunit of TFIIH and nucleotide excision repair factor 3 complexes	-0.59547	0.001666	down
PAS_chr4_0750	Hypothetical protein	-0.66646	0.030879	down
PAS_chr4_0756	Mitochondrial protein kinase	-0.60415	0.001337	down
PAS_chr4_0791	Beta subunit of geranylgeranyltransferase type I	-0.90407	0.00095	down
PAS_chr4_0793	Hypothetical protein	-0.68666	0.001665	down
PAS_chr4_0803	Nucleolar protein involved in the assembly of the large ribosomal subunit	-0.75349	0.005853	down
PAS_chr4_0827	Hypothetical protein	-0.7801	6.31E-07	down
PAS_chr4_0837	Hypothetical protein	-1.00444	6.29E-08	down
PAS_chr4_0854	Hypothetical protein	-0.85112	0.00224	down
PAS_chr4_0856	Component, with Yta12p, of the mitochondrial inner membrane m-AAA protease	-0.65288	9.34E-11	down
PAS_chr4_0858	Hypothetical protein	-0.70535	9.04E-08	down
PAS_chr4_0862	Hypothetical protein	-0.6069	0.008513	down
PAS_chr4_0867	Hypothetical protein	-0.94004	4.22E-08	down
PAS_chr4_0869	Non-essential protein of unknown function	-0.67366	0.002079	down
PAS_chr4_0872	Hypothetical protein	-0.9131	0.000692	down
PAS_chr4_0876	Hypothetical protein	-0.6168	0.005431	down
PAS_chr4_0901	Ribosomal RNA-processing protein	-1.06723	5.23E-13	down
PAS_chr4_0942	hypothetical protein	-0.67101	0.000521	down
PAS_chr4_0957	hypothetical protein	-0.63156	0.01327	down
PAS_chr4_0960	hypothetical protein	-0.66718	6.80E-12	down
PAS_chr4_0965	hypothetical protein	-0.70259	0.000725	down
PAS_chr4_0966	hypothetical protein	-0.67441	0.000342	down
PAS_chr4_0986	hypothetical protein	-0.63244	5.05E-09	down
PAS_chr4_0990	hypothetical protein	-0.8912	8.98E-17	down
PAS_chr4_0992	hypothetical protein	-0.67236	4.35E-05	down
PAS_chr4_1001	hypothetical protein	-0.96833	0.011037	down

Supplementary Table S2 Total enrichment results of biological pathways via KEGG

Num	Description	Padjust	Gene_names
60	Ribosome	6.45E-08	PAS_chr3_0290;PAS_chr3_1057;PAS_chr1-1_0345;PAS_chr1-4_0353;PAS_chr1-4_0352;PAS_chr2-1_0087;PAS_chr2-1_0086;PAS_chr3_0335;PAS_chr2-1_0728;PAS_chr4_0982;PAS_chr1-4_0589;PAS_chr2-2_0054;PAS_chr2-1_0683;PAS_FragB_0037;PAS_chr2-2_0229;PAS_chr1-3_0300;PAS_chr2-2_0326;PAS_chr4_0246;PAS_chr1-3_0115;PAS_chr1-3_0034;PAS_chr1-1_0183;PAS_chr4_0041;PAS_chr1-4_0422;PAS_chr2-1_0481;PAS_chr4_0107;PAS_chr1-1_0189;PAS_chr1-4_0471;PAS_chr3_0596;PAS_chr1-4_0148;PAS_chr1-1_0296;PAS_chr4_0292;PAS_chr4_0211;PAS_chr2-1_0362;PAS_chr1-1_0439;PAS_chr2-1_0482;PAS_chr4_0799;PAS_c131_0011;PAS_chr4_0813;PAS_chr1-4_0490;PAS_chr4_0412;PAS_chr4_0413;PAS_chr3_0946;PAS_chr4_0414;PAS_chr4_0456;PAS_chr3_0762;PAS_chr3_1200;PAS_chr2-2_0109;PAS_chr4_0139;PAS_chr1-1_0216;PAS_chr4_0524;PAS_chr1-1_0076;PAS_chr2-2_0257;PAS_chr3_0091;PAS_chr1-3_0068;PAS_chr3_0722;PAS_chr4_0131;PAS_chr2-1_0658;PAS_chr2-1_0022;PAS_chr2-1_0634;PAS_chr1-1_0219
16	Methane metabolism	0.001309	PAS_chr3_0826;PAS_chr3_0834;PAS_chr1-1_0427;PAS_chr4_0416;PAS_chr4_0821;PAS_chr3_0832;PAS_chr4_0285;PAS_chr4_0587;PAS_chr3_0566;PAS_chr3_1028;PAS_chr3_0868;PAS_chr1-1_0072;PAS_chr3_0932;PAS_chr3_0082;PAS_chr3_0867;PAS_chr2-1_0657
16	Alanine, aspartate and glutamate metabolism	0.010594	PAS_chr4_0416;PAS_chr2-2_0329;PAS_chr3_0591;PAS_chr1-1_0107;PAS_chr3_0799;PAS_chr1-3_0024;PAS_chr4_0613;PAS_chr3_0743;PAS_chr4_0677;PAS_chr1-1_0200;PAS_chr3_0675;PAS_chr4_0785;PAS_chr4_0974;PAS_chr1-4_0227;PAS_chr4_0138;PAS_chr3_0482
18	Glycine, serine and threonine metabolism	0.01075	PAS_chr3_0826;PAS_chr1-4_0049;PAS_chr1-1_0427;PAS_chr4_0416;PAS_chr4_0112;PAS_FragB_0009;PAS_chr4_0285;PAS_chr4_0587;PAS_chr3_0566;PAS_chr3_0349;PAS_chr4_0150;PAS_chr1-4_0489;PAS_chr2-1_0657;PAS_chr1-4_0243;PAS_chr4_0938;PAS_chr3_0634;PAS_chr2-1_0679;PAS_chr3_0899
33	Oxidative phosphorylation	0.010796	PAS_chr3_0585;PAS_chr3_0808;PAS_chr1-1_0002;PAS_chr3_0576;PAS_chr3_0997;PAS_chr1-4_0487;PAS_chr4_0733;PAS_chr2-2_0227;PAS_chr3_0460;PAS_chr3_0615;PAS_chr4_0990;PAS_chr1-1_0309;PAS_chr2-1_0687;PAS_chr1-4_0445;PAS_chr1-3_0070;PAS_chr4_0120;PAS_chr2-2_0265;PAS_chr1-4_0371;PAS_chr2-2_0430;PAS_chr2-2_0462;PAS_chr3_0819;PAS_chr3_1040;PAS_chr2-1_0451;PAS_chr2-1_0363;PAS_chr1-1_0099;PAS_chr2-2_0266;PAS_chr3_0059;PAS_chr1-3_0194;PAS_chr1-1_0484;PAS_chr1-3_0028;PAS_chr2-2_0165;PAS_chr1-1_0191;PAS_chr4_0520
13	Glyoxylate and dicarboxylate metabolism	0.012365	PAS_chr2-2_0131;PAS_chr4_0593;PAS_chr4_0587;PAS_FragB_0009;PAS_chr1-4_0304;PAS_chr1-1_0475;PAS_chr1-3_0104;PAS_chr4_0416;PAS_chr2-1_0238;PAS_chr3_0932;PAS_chr4_0785;PAS_chr4_0938;PAS_chr4_0815
14	Citrate cycle (TCA cycle)	0.0124	PAS_chr2-1_0120;PAS_chr1-4_0593;PAS_chr3_0831;PAS_chr1-3_0104;PAS_chr3_0647;PAS_chr1-1_0475;PAS_chr4_0733;PAS_chr4_0580;PAS_chr2-2_0294;PAS_chr2-1_0238;PAS_chr1-1_0233;PAS_chr4_0815;PAS_chr1-1_0050;PAS_chr1-4_0487
8	Thiamine metabolism	0.024381	PAS_chr3_0648;PAS_chr4_0065;PAS_chr2-1_0111;PAS_chr1-4_0151;PAS_chr3_0257;PAS_chr2-2_0084;PAS_chr3_0842;PAS_chr3_0843
18	Glycolysis / Gluconeogenesis	0.027008	PAS_chr3_0826;PAS_chr3_0082;PAS_chr1-1_0427;PAS_chr1-4_0264;PAS_chr3_0006;PAS_chr2-1_0472;PAS_chr4_0624;PAS_chr1-4_0292;PAS_chr1-1_0050;PAS_chr1-4_0593;PAS_chr3_0868;PAS_chr2-2_0294;PAS_chr1-1_0072;PAS_chr2-1_0437;PAS_chr4_0043;PAS_chr2-1_0853;PAS_chr3_1028;PAS_chr3_0951
7	Biosynthesis of unsaturated fatty acids	0.061852	PAS_chr2-1_0072;PAS_chr4_0368;PAS_chr4_0780;PAS_chr4_0743;PAS_chr3_0222;PAS_chr4_0052;PAS_chr3_0236
14	Cysteine and methionine metabolism	0.271326	PAS_chr3_0404;PAS_chr1-4_0049;PAS_chr4_0815;PAS_chr3_0890;PAS_chr2-1_0358;PAS_chr3_0566;PAS_chr4_0280;PAS_chr1-4_0489;PAS_chr4_0974;PAS_chr1-1_0200;PAS_chr2-1_0657;PAS_chr4_0330;PAS_chr2-1_0238;PAS_chr4_0150
4	Nitrogen metabolism	0.279164	PAS_chr1-1_0107;PAS_chr4_0578;PAS_chr2-1_0037;PAS_chr4_0785
8	Arginine biosynthesis	0.303115	PAS_chr1-1_0107;PAS_chr1-1_0127;PAS_chr1-1_0200;PAS_chr3_0482;PAS_chr4_0785;PAS_chr4_0974;PAS_chr3_0623;PAS_chr3_0176
10	Arginine and proline metabolism	0.375576	PAS_chr4_0823;PAS_chr1-3_0269;PAS_chr3_0410;PAS_chr4_0398;PAS_chr4_0665;PAS_chr2-1_0853;PAS_chr1-1_0200;PAS_chr4_0974;PAS_chr4_0043;PAS_chr1-4_0227
9	Pentose phosphate pathway	0.384813	PAS_chr3_0277;PAS_chr1-4_0150;PAS_chr2-2_0338;PAS_chr1-4_0264;PAS_chr2-2_0337;PAS_chr4_0212;PAS_chr3_0868;PAS_chr1-1_0072;PAS_chr1-4_0669
6	Fatty acid degradation	0.387705	PAS_chr2-1_0472;PAS_chr1-4_0304;PAS_chr2-1_0853;PAS_chr1-4_0663;PAS_chr4_0043;PAS_chr3_1028
6	Lysine biosynthesis	0.387705	PAS_chr1-4_0049;PAS_chr4_0150;PAS_chr4_0795;PAS_chr2-2_0168;PAS_chr1-4_0421;PAS_chr3_0528
4	Fatty acid elongation	0.422519	PAS_chr4_0780;PAS_chr4_0368;PAS_chr3_0236;PAS_chr3_0222
5	Valine, leucine and	0.431546	PAS_chr1-4_0243;PAS_chr2-1_0864;PAS_chr3_0039;PAS_chr1-1_0432;PAS_chr2-1_0415

	isoleucine biosynthesis		
5	Butanoate metabolism	0.431546	PAS_chr2-1_0191;PAS_chr4_0677;PAS_chr1-4_0304;PAS_chr2-1_0864;PAS_chr1-3_0024
15	Pyruvate metabolism	0.446508	PAS_chr3_0006;PAS_chr2-1_0472;PAS_chr1-4_0304;PAS_chr3_0647;PAS_chr1-1_0050;PAS_chr1-4_0593;PAS_chr4_0336;PAS_chr2-2_0294;PAS_chr2-1_0238;PAS_chr2-1_0415;PAS_chr1-4_0421;PAS_chr4_0815;PAS_chr2-1_0853;PAS_chr3_1028;PAS_chr4_0043
4	Selenocompound metabolism	0.46835	PAS_chr1-4_0489;PAS_chr2-1_0396;PAS_chr2-1_0358;PAS_chr1-4_0253
35	Biosynthesis of cofactors	0.476035	PAS_chr2-1_0273;PAS_chr4_0065;PAS_chr2-1_0111;PAS_chr1-1_0169;PAS_chr4_0613;PAS_chr2-2_0084;PAS_chr2-2_0329;PAS_chr4_0138;PAS_chr1-3_0016;PAS_chr3_0566;PAS_chr1-4_0198;PAS_chr2-2_0303;PAS_chr3_0954;PAS_chr3_0842;PAS_chr3_0843;PAS_chr1-4_0678;PAS_chr1-3_0194;PAS_chr3_0257;PAS_chr4_0043;PAS_chr4_0940;PAS_chr3_0648;PAS_chr4_0823;PAS_chr4_0587;PAS_chr4_0418;PAS_chr4_0981;PAS_chr3_1115;PAS_chr3_1037;PAS_chr2-1_0853;PAS_chr2-2_0053;PAS_chr3_0799;PAS_chr3_0167;PAS_chr3_0870;PAS_chr3_1012;PAS_chr2-2_0059;PAS_chr2-1_0593
7	Lysine degradation	0.484464	PAS_chr2-2_0152;PAS_chr1-3_0024;PAS_chr2-1_0853;PAS_chr4_0043;PAS_chr1-1_0129;PAS_chr1-4_0304;PAS_chr3_0528
7	SNARE interactions in vesicular transport	0.484464	PAS_chr3_0989;PAS_chr1-1_0481;PAS_chr2-2_0453;PAS_chr1-4_0462;PAS_chr1-4_0024;PAS_chr3_0048;PAS_chr1-1_0246
10	Fructose and mannose metabolism	0.485521	PAS_chr4_0341;PAS_chr4_0988;PAS_chr4_0624;PAS_chr3_1115;PAS_chr3_0868;PAS_chr1-1_0072;PAS_chr2-2_0053;PAS_chr3_0870;PAS_chr2-1_0870;PAS_chr3_0951
8	Pantothenate and CoA biosynthesis	0.518067	PAS_chr4_0823;PAS_chr1-1_0432;PAS_chr1-4_0198;PAS_chr4_0793;PAS_chr2-1_0853;PAS_chr3_1012;PAS_chr2-1_0864;PAS_chr4_0043
4	Pentose and glucuronate interconversions	0.521248	PAS_chr2-2_0019;PAS_chr4_0572;PAS_chr3_0086;PAS_chr3_0744
2	C5-Branched dibasic acid metabolism	0.526696	PAS_chr3_0039;PAS_chr2-1_0864
12	Proteasome	0.543227	PAS_chr3_1069;PAS_chr4_0387;PAS_chr4_0299;PAS_chr3_0457;PAS_chr3_0699;PAS_chr4_0736;PAS_chr1-3_0195;PAS_chr2-2_0341;PAS_chr2-1_0597;PAS_chr3_0748;PAS_chr4_0668;PAS_chr1-1_0079
7	Protein export	0.611889	PAS_chr2-1_0379;PAS_chr1-4_0134;PAS_chr1-1_0274;PAS_chr1-4_0629;PAS_chr1-4_0210;PAS_chr1-4_0577;PAS_chr1-3_0202
8	Amino sugar and nucleotide sugar metabolism	0.642909	PAS_chr1-4_0264;PAS_chr1-1_0393;PAS_chr4_0624;PAS_chr3_1115;PAS_chr1-1_0067;PAS_chr4_0737;PAS_chr2-2_0053;PAS_chr3_0870
6	Terpenoid backbone biosynthesis	0.655526	PAS_chr2-1_0191;PAS_chr4_0834;PAS_chr1-4_0304;PAS_chr3_0130;PAS_chr1-3_0192;PAS_chr1-4_0429
2	Mannose type O-glycan biosynthesis	0.671748	PAS_chr2-1_0212;PAS_chr2-1_0256
2	Monobactam biosynthesis	0.671748	PAS_chr1-4_0253;PAS_chr4_0150
23	Spliceosome	0.672132	PAS_chr3_0325;PAS_chr1-3_0182;PAS_chr3_1252;PAS_chr3_0400;PAS_FragB_0004;PAS_chr4_0552;PAS_chr3_1041;PAS_chr1-3_0256;PAS_chr4_0670;PAS_chr3_0731;PAS_chr1-4_0686;PAS_chr1-1_0014;PAS_chr2-1_0455;PAS_chr2-1_0450;PAS_chr1-4_0614;PAS_chr4_0201;PAS_chr4_0942;PAS_chr2-1_0818;PAS_chr3_0642;PAS_chr3_0644;PAS_chr4_0538;PAS_chr2-1_0187;PAS_chr2-2_0424
3	Cyanoamino acid metabolism	0.689058	PAS_chr4_0587;PAS_chr2-1_0037;PAS_chr3_0591
5	Tyrosine metabolism	0.692816	PAS_chr2-1_0472;PAS_chr4_0974;PAS_chr1-1_0200;PAS_chr3_1028;PAS_chr1-3_0024
5	Valine, leucine and isoleucine degradation	0.692816	PAS_chr2-1_0191;PAS_chr4_0677;PAS_chr1-4_0304;PAS_chr2-1_0853;PAS_chr4_0043
9	Basal transcription factors	0.915397	PAS_chr1-4_0318;PAS_chr2-1_0838;PAS_chr1-1_0012;PAS_chr2-1_0277;PAS_chr3_0737;PAS_chr4_0745;PAS_chr4_0518;PAS_chr2-1_0043;PAS_chr2-1_0142
3	Vitamin B6	0.920535	PAS_chr2-1_0273;PAS_chr4_0981;PAS_chr3_0566

	metabolism		
5	Tryptophan metabolism	0.943279	PAS_chr1-4_0304;PAS_chr2-2_0131;PAS_chr2-1_0853;PAS_chr2-1_0037;PAS_chr4_0043
1	Carbapenem biosynthesis	0.990189	PAS_chr4_0665
5	Homologous recombination	0.99417	PAS_chr3_0224;PAS_chr4_0299;PAS_chr1-1_0242;PAS_FragB_0039;PAS_chr2-1_0153
4	Nicotinate and nicotinamide metabolism	0.994391	PAS_chr1-1_0132;PAS_chr1-1_0374;PAS_chr1-3_0130;PAS_chr1-3_0024
4	Steroid biosynthesis	0.998876	PAS_chr3_0053;PAS_chr1-4_0367;PAS_chr4_0198;PAS_chr1-4_0604
4	Porphyrin and chlorophyll metabolism	0.998876	PAS_chr4_0418;PAS_chr2-2_0293;PAS_chr3_0954;PAS_chr1-3_0194
8	Meiosis - yeast	0.999831	PAS_chr2-1_0061;PAS_chr3_0540;PAS_chr3_1078;PAS_chr2-1_0276;PAS_chr3_0477;PAS_chr4_0604;PAS_chr4_0603;PAS_FragB_0028
3	Ascorbate and aldarate metabolism	1	PAS_chr4_0043;PAS_chr2-1_0853;PAS_chr2-1_0197
9	Phagosome	1	PAS_chr3_0989;PAS_chr3_1040;PAS_chr1-3_0113;PAS_chr2-2_0227;PAS_chr1-4_0629;PAS_chr4_0990;PAS_chr1-1_0309;PAS_chr1-3_0202;PAS_chr2-1_0687
5	beta-Alanine metabolism	1	PAS_chr4_0677;PAS_chr4_0043;PAS_chr2-1_0853;PAS_chr4_0823;PAS_chr1-4_0198
19	Protein processing in endoplasmic reticulum	1	PAS_chr4_0436;PAS_chr1-4_0130;PAS_chr1-3_0202;PAS_chr1-1_0367;PAS_chr3_0731;PAS_chr3_0480;PAS_chr1-4_0629;PAS_chr4_0552;PAS_chr4_0991;PAS_chr1-3_0174;PAS_chr4_0606;PAS_chr1-1_0180;PAS_chr2-1_0421;PAS_chr2-1_0042;PAS_chr1-1_0237;PAS_chr1-3_0289;PAS_chr2-2_0015;PAS_chr2-1_0753;PAS_chr1-4_0685
3	Riboflavin metabolism	1	PAS_chr3_0167;PAS_chr3_1037;PAS_chr1-4_0151
7	N-Glycan biosynthesis	1	PAS_chr3_0552;PAS_chr3_0999;PAS_chr4_0264;PAS_chr2-1_0753;PAS_chr2-1_0759;PAS_chr1-1_0459;PAS_chr1-4_0685
7	Pyrimidine metabolism	1	PAS_chr3_0262;PAS_chr1-1_0132;PAS_chr3_0799;PAS_chr3_0349;PAS_chr4_0138;PAS_chr2-2_0059;PAS_chr2-1_0593
2	Other types of O-glycan biosynthesis	1	PAS_chr2-1_0256;PAS_chr2-1_0212
5	Propanoate metabolism	1	PAS_chr4_0677;PAS_chr1-4_0304;PAS_chr2-1_0313;PAS_chr3_0831;PAS_chr4_0336
5	Phosphatidylinositol signaling system	1	PAS_chr4_0700;PAS_chr1-1_0005;PAS_chr1-4_0617;PAS_chr1-1_0457;PAS_chr2-1_0758
5	Phenylalanine, tyrosine and tryptophan biosynthesis	1	PAS_chr4_0974;PAS_chr3_0634;PAS_chr1-1_0200;PAS_chr2-1_0679;PAS_chr2-1_0637
18	Endocytosis	1	PAS_chr2-2_0087;PAS_chr3_0150;PAS_chr2-1_0572;PAS_chr3_1165;PAS_chr2-1_0112;PAS_c131_0006;PAS_chr4_0635;PAS_chr4_0552;PAS_chr3_0238;PAS_chr1-3_0288;PAS_chr1-1_0457;PAS_chr3_0731;PAS_chr1-4_0023;PAS_chr1-3_0184;PAS_chr1-4_0340;PAS_chr3_0458;PAS_chr3_0312;PAS_chr1-1_0034
1	Phosphonate and phosphinate metabolism	1	PAS_chr2-2_0401
3	Sulfur metabolism	1	PAS_chr1-4_0253;PAS_chr4_0369;PAS_chr4_0330
12	Purine metabolism	1	PAS_chr1-4_0253;PAS_chr1-4_0264;PAS_chr2-2_0329;PAS_chr2-1_0111;PAS_chr2-1_0101;PAS_chr1-1_0132;PAS_chr1-4_0450;PAS_chr1-3_0191;PAS_chr3_1138;PAS_chr3_0257;PAS_chr4_0613;PAS_chr2-2_0059
5	Inositol phosphate metabolism	1	PAS_chr4_0700;PAS_chr1-4_0617;PAS_chr3_0951;PAS_chr1-1_0457;PAS_chr2-2_0113
2	Ubiquinone and other terpenoid-quinone biosynthesis	1	PAS_chr1-1_0169;PAS_chr2-2_0303
3	Sphingolipid metabolism	1	PAS_chr3_0597;PAS_chr3_0399;PAS_chr4_0866
10	Peroxisome	1	PAS_chr2-2_0131;PAS_chr4_0416;PAS_chr3_0099;PAS_chr2-1_0502;PAS_chr3_1073;PAS_chr2-1_0504;PAS_chr4_0786;PAS_chr1-4_0663;PAS_chr3_0043;PAS_chr1-1_0233

2	Non-homologous end-joining	1	PAS_chr1-4_0116;PAS_chr1-4_0633
5	Starch and sucrose metabolism	1	PAS_chr3_0781;PAS_chr1-4_0426;PAS_chr1-4_0264;PAS_chr4_0847;PAS_chr4_0624
2	Galactose metabolism	1	PAS_chr1-4_0264;PAS_chr4_0624
3	Folate biosynthesis	1	PAS_chr4_0940;PAS_chr2-2_0084;PAS_chr1-4_0678
1	Biotin metabolism	1	PAS_chr1-3_0016
3	Base excision repair	1	PAS_chr2-2_0280;PAS_chr1-4_0633;PAS_chr1-3_0055
6	Longevity regulating pathway - multiple species	1	PAS_chr2-2_0131;PAS_chr4_0552;PAS_chr4_0786;PAS_chr3_0731;PAS_chr1-1_0374;PAS_chr2-1_0324
1	Ether lipid metabolism	1	PAS_chr2-2_0063
11	RNA degradation	1	PAS_chr4_0229;PAS_chr2-1_0266;PAS_chr4_0158;PAS_chr4_0224;PAS_chr3_0753;PAS_chr3_1042;PAS_chr1-3_0256;PAS_chr3_0603;PAS_chr3_0956;PAS_chr3_0082;PAS_chr1-1_0014
4	Glutathione metabolism	1	PAS_chr3_0277;PAS_chr1-1_0433;PAS_chr1-1_0233;PAS_chr3_0906
5	Various types of N-glycan biosynthesis	1	PAS_chr3_0620;PAS_chr1-4_0037;PAS_chr2-1_0753;PAS_chr2-1_0759;PAS_chr1-4_0685
5	Glycerolipid metabolism	1	PAS_chr2-1_0279;PAS_chr4_0043;PAS_chr2-1_0853;PAS_chr3_0006;PAS_chr4_0938
2	Sulfur relay system	1	PAS_chr1-4_0547;PAS_chr2-2_0373
2	One carbon pool by folate	1	PAS_chr2-2_0145;PAS_chr4_0587
2	Histidine metabolism	1	PAS_chr4_0043;PAS_chr2-1_0853
2	Phenylalanine metabolism	1	PAS_chr4_0974;PAS_chr1-1_0200
8	Ubiquitin mediated proteolysis	1	PAS_chr3_1078;PAS_chr2-1_0369;PAS_chr3_0359;PAS_chr3_0477;PAS_chr4_0436;PAS_chr3_0709;PAS_chr3_0722;PAS_FragB_0055
1	Hippo signaling pathway - multiple species	1	PAS_chr1-4_0419
4	RNA polymerase	1	PAS_chr3_0244;PAS_chr2-2_0434;PAS_chr1-4_0359;PAS_chr4_0925
12	Ribosome biogenesis in eukaryotes	1	PAS_chr1-4_0403;PAS_chr2-1_0387;PAS_chr2-1_0449;PAS_chr2-1_0781;PAS_chr3_0112;PAS_chr4_0278;PAS_chr1-4_0640;PAS_chr2-1_0648;PAS_chr3_1041;PAS_chr3_0885;PAS_chr1-1_0377;PAS_chr3_0681
7	mRNA surveillance pathway	1	PAS_chr2-2_0133;PAS_chr1-1_0015;PAS_chr2-1_0414;PAS_chr2-1_0528;PAS_chr1-3_0074;PAS_chr2-1_0232;PAS_chr2-1_0233
1	Fatty acid biosynthesis	1	PAS_chr1-3_0016
10	Nucleocytoplasmic transport	1	PAS_chr4_0719;PAS_chr2-1_0617;PAS_chr2-1_0528;PAS_chr3_0431;PAS_chr2-1_0449;PAS_chr4_0551;PAS_chr3_0312;PAS_FragB_0052;PAS_chr3_0400;PAS_chr1-3_0074
18	Cell cycle - yeast	1	PAS_chr2-1_0061;PAS_chr3_0540;PAS_chr4_0436;PAS_chr2-1_0276;PAS_chr2-1_0088;PAS_chr2-1_0627;PAS_chr3_0477;PAS_chr4_0604;PAS_chr3_1078;PAS_chr2-1_0092;PAS_chr3_0968;PAS_chr4_0603;PAS_chr4_0129;PAS_FragB_0028;PAS_chr3_1186;PAS_chr1-4_0419;PAS_FragD_0025;PAS_chr2-2_0298
5	Nucleotide excision repair	1	PAS_chr2-1_0043;PAS_chr4_0436;PAS_chr2-1_0629;PAS_chr4_0745;PAS_chr2-1_0702
5	Aminoacyl-tRNA biosynthesis	1	PAS_chr2-1_0396;PAS_chr2-2_0238;PAS_chr1-4_0463;PAS_chr2-2_0209;PAS_chr3_1110
14	MAPK signaling pathway - yeast	1	PAS_chr3_0299;PAS_chr2-2_0131;PAS_chr4_0584;PAS_chr2-2_0111;PAS_chr4_0986;PAS_chr2-1_0112;PAS_chr2-1_0117;PAS_chr3_0968;PAS_chr1-3_0149;PAS_chr4_0336;PAS_chr1-1_0201;PAS_chr1-1_0457;PAS_chr4_0123;PAS_chr3_0150
1	ABC	1	PAS_chr2-1_0613

	transporters		
3	DNA replication	1	PAS_chr1-1_0049;PAS_chr1-4_0633;PAS_chr1-1_0474
3	Glycerophospholipid metabolism	1	PAS_chr2-2_0401;PAS_chr2-2_0111;PAS_chr2-2_0063
3	Mitophagy - yeast	1	PAS_chr3_0263;PAS_chr1-4_0614;PAS_chr1-1_0457
1	Autophagy - other	1	PAS_chr1-4_0119
9	Autophagy - yeast	1	PAS_chr3_1087;PAS_chr2-1_0672;PAS_chr3_0210;PAS_chr3_0238;PAS_chr1-4_0606;PAS_chr1-4_0119;PAS_chr2-1_0574;PAS_chr1-4_0548;PAS_chr3_0458
